# The Role of Magnetic Resonance Spectroscopy (MRS), Diffusion‐Tensor‐Imaging (DTI) and Structural MRI in the Alzheimer's Disease and Mild Cognitive Impairment Diagnosis: A Review

**DOI:** 10.1002/jmri.70296

**Published:** 2026-03-19

**Authors:** Valentina Zecca, Gianmauro Palombelli, Nicola Vanacore, Rossella Canese

**Affiliations:** ^1^ MRI Unit, Core Facilities, Istituto Superiore di Sanità Rome Italy; ^2^ Department of Basic and Applied Sciences for Engineering University of Rome Sapienza Rome Italy; ^3^ National Centre for Disease Prevention and Health Promotion, Istituto Superiore di Sanità Rome Italy

**Keywords:** Alzheimer's disease, dementia, DTI, MRI, MRS

## Abstract

Alzheimer's disease (AD) is one of the most common neurological disorders affecting older adults, with approximately 7.2 million cases only in the United States. This number is projected to increase to 13.8 million in the United States by 2060, leading to increased expenditures for healthcare, long‐term care and hospice services. Consequently, great emphasis is placed on prevention and the development of early diagnosis techniques, which can lead to timely treatment and the prevention of the consequences of full‐blown disease. In this review, we analyze the potential diagnostic value of biomarkers derived from a multimodal approach based on magnetic resonance spectroscopy, diffusion tensor imaging, and magnetic resonance imaging, capable of detecting metabolic, microstructural, and anatomical changes, respectively, that precede the cognitive and behavioral changes observed in AD by years. The primary aim is to evaluate whether the combined and complementary use of these methods can identify early biomarkers useful for recognizing AD in its early stages, predicting progression from MCI to AD, supporting patient stratification, and monitoring cognitive decline or response to treatment. We identified regions more susceptible to metabolic alterations (PCC and hippocampus) and trajectories of structural brain alterations (atrophy or diffusivity abnormalities). The assessment of such imaging biomarkers may serve as the foundation for future prospective studies aimed at developing differential diagnostic methods, a crucial goal within the broader context of dementias, by adopting standardized multimodal MRI protocols.

**Evidence Level:**

3.

**Technical Efficacy:**

Stage 1.

## Highlights on the Usefulness of Multimodal MRI in Diagnosis, Early Diagnosis and Treatment Monitoring of AD

- Multimodal MRI can be valuable for early diagnosis, given that metabolic alterations (via Magnetic Resonance Spectroscopy, MRS), microstructural modifications (via Diffusion Tensor Imaging, DTI), and volumetric changes (via Magnetic Resonance Imaging, MRI).
- These alterations can be detected years before the onset of clinical symptoms.
- These are non‐invasive tools that can support clinicians in making a diagnosis, promote timely intervention aimed at slowing or preventing progression to full‐blown disease, and guide the adoption of personalized therapeutic strategies.
- Furthermore, these imaging modalities provide a valuable means to assess the potential efficacy of both pharmacological and non‐pharmacological treatments.

## Plain Language Summary

Alzheimer's disease affects millions of older adults, and its numbers are expected to rise sharply in the coming decades. This review explores whether combining different MRI‐based techniques can detect early brain changes—metabolic, structural, and anatomical—that appear years before memory problems. The findings highlight key vulnerable areas, such as the hippocampus and posterior cingulate cortex, and suggest that multimodal imaging could help identify early Alzheimer's, predict progression from mild cognitive impairment, and improve patient monitoring.

## 1 Introduction

It is estimated that about 7.2 million Americans had Alzheimer's disease in 2025, and a similar number is estimated to have Mild Cognitive Impairment (MCI) [1]. However, the number of individuals affected by dementia is expected to increase in the coming years due to the rising average age of the global population. According to the Alzheimer Europe Organization, based on the population of 27 European countries, it is estimated that about 9 million EU citizens are affected by Alzheimer's disease in 2025. By 2050, the EU population is projected to reach 506 million, with approximately 14.3 million people living with AD (www.alzheimer‐europe.org). This scenario—characterized by the significant social and economic impact of dementia—has led to the development of national guidelines providing recommendations aimed at ensuring the appropriateness of clinical practices for patients and caregivers in the context of diagnosis, treatment, and care.

The new diagnostic criteria for dementia are promoted by the International Working Group (IWG) and the National Institute on Aging (NIA‐AA) [2, 3] and refer to two conditions considered risk factors: MCI (to identify early stages of dementia [4]) and SCD (subjective cognitive decline). For MCI, the classic Petersen criteria (1999–2001–2004) require preserved functional abilities and the absence of dementia [5, 6]. The earliest criteria were the 1984 NINCDS‐ADRDA criteria [7], followed by the 1994 American Psychiatric Association DSM‐IV criteria, which defined dementia on a clinical basis.

It is important to note that a substantial proportion of individuals with MCI or SCD will never progress to dementia, and some may even return to a normal cognitive profile. Furthermore, it has been estimated that the lifetime risk of developing dementia for a 60‐year‐old man and woman with amyloidosis is only 23% and 31%, respectively. In fact, many individuals who test positive for MCI/AD biomarkers will likely not exhibit significant cognitive or functional decline over time. New biomarkers could help distinguish between individuals with stable versus progressive MCI.

Currently, in clinical practice, neurologists primarily use neuropsychological assessment tools such as the MMSE, combined with tests for tau protein in cerebrospinal fluid and/or evaluations of amyloid‐PET or tau‐PET, as well as assessments of cerebral atrophy through structural MRI.

Recently, Tideman and colleagues demonstrated that a brief, self‐administered digital cognitive test battery can detect cognitive impairment and, when combined with a blood test, accurately identify clinical AD in primary care settings [8]. However, no biomarker has been consistently associated with the clinical trajectory of the disease, particularly in the preclinical and prodromal phases. The implementation of new diagnostic strategies aimed at identifying early biomarkers capable of detecting preclinical forms of AD is therefore essential. This would allow clinicians to intervene promptly with therapeutic strategies to slow disease progression. A significant number of clinical studies are currently evaluating new approaches to refine early AD diagnosis. In this review, we provide an overview of recent MR‐based studies to determine whether the use of Magnetic Resonance Spectroscopy (MRS), Diffusion Tensor Imaging (DTI), and structural MRI can offer complementary information to the “Comprehensive Geriatric Assessment (CGA),” a multidimensional and multidisciplinary process designed to optimize diagnostic accuracy, improve medical treatment, enhance quality of life, and organize long‐term personalized management for older adults [9].

### 1.1 Targeting MCI to Reduce the Burden of Dementia

In this study, we focused on AD (the most extensively studied form of dementia, with a significantly higher number of articles indexed in PubMed compared to other types of dementia) and MCI. In fact, in recent years, research interest has increasingly shifted toward MCI, which represents a preclinical transitional stage between healthy aging and dementia, with particular focus on amnestic MCI (aMCI). In aMCI, memory loss predominates and is classified into two subtypes: single‐domain aMCI (S‐aMCI) and multiple‐domain aMCI (M‐aMCI). S‐aMCI is characterized primarily by isolated memory deficits, whereas M‐aMCI involves memory deficits accompanied by impairments in other cognitive domains such as language, executive functions, and attention. M‐aMCI carries a higher risk of conversion to AD compared to S‐aMCI.

A growing body of research has emphasized the importance of intervening in MCI to delay progression to dementia, potentially reducing the incidence of AD and significantly alleviating the social and economic burden of dementia. It is essential to refine clinical criteria, estimate its prevalence and prognosis, and explore strategies for prevention, early and differential diagnosis, and therapeutic intervention.

### 1.2 Multimodal MRI: MRS, DTI and Structural MRI

We evaluate the diagnostic role of MRS, DTI and structural MRI in AD and in its preclinical phase (MCI). The focus is AD, the most prevalent form of dementia, whose main risk factors include aging and mitochondrial dysfunctions, both of which contribute to neurodegenerative processes such as synaptic and neuronal loss, disruption of energy metabolism, and increased production of reactive oxygen species (ROS) [10].

MRS is a non‐invasive MR tool that allows us to characterize and quantify oxidative stress and neuroinflammation associated with bioenergetic and metabolic abnormalities in MCI and AD in specific brain regions, observing a reduction in N‐acetylaspartate (NAA) and an increase in myo‐inositol (mI).

NAA is one of the most studied metabolites in the brain by MRS, because its methyl group gives rise to a prominent signal resonating at 2.02 ppm. It is present in normal brain at 7.5–17 mM concentration [11] and could represent an indicator of neuronal integrity and viability as it is synthesized mainly in neuronal mitochondria. It has also been found in immature oligodendrocytes, the cells responsible for myelin production in the central nervous system. Oligodendrocytes utilize the acetate derived from NAA for myelin synthesis. NAA decreases in AD with longitudinal variations over time, that is, with disease progression. The evidence gathered in this review leads to the conclusion that NAA reduction is associated with tau accumulation, early neurodegenerative processes, cognitive decline, and disease severity. These are the reasons why NAA could be used as a marker to assess the progress of disease‐modifying therapies.

mI is another important compound detectable by MRS, primarily found in astrocytes with a characteristic signal resonating at 3.56 ppm, present in normal brain at 4–8 mM concentration [11]. It plays a key role in phospholipid metabolism and neuronal signaling, functioning as an osmolyte and as a precursor of second messengers. Being into the astrocytes, which are the glial cells most involved in the inflammatory response in the CNS, myo‐inositol increase is due to astrocytes' activation (reactive gliosis) during inflammatory processes. We could observe its increase in several neurodegenerative and inflammatory/demyelinating diseases [12].

However, both the reduction in NAA and the increase in mI are not specific to AD. Indeed, the reduction in NAA occurs in several other neurodegenerative diseases (amyotrophic lateral sclerosis, multiple sclerosis, Huntington's Disease) or in other pathologies such as traumatic brain injury, epilepsy, and brain tumors.

DTI is a MR tool that allows us to observe the microstructure of white and gray matter and estimate the movement of water molecules at each voxel. In white matter (WM), water tends to move along the direction of the nerve fibers (anisotropic diffusion) because the myelin and axons restrict movement perpendicular to the fibers. DTI allows us to identify damage to WM integrity caused by AD even in its early stages [13].

DTI uses the direction and extent of diffusion to obtain information about possible WM damage through parameters such as: Fractional Anisotropy (FA), which measures whether diffusion is directional (high FA value indicates well‐organized fibers); Mean Diffusivity (MD), which indicates the entity of diffusion without taking care of the direction (high MD value indicates tissue damage, edema, or neurodegeneration); Axial Diffusivity (AD), which measures diffusion along the main axis of the fiber (its reduction indicates axonal damage); and Radial Diffusivity (RD), which measures diffusion perpendicular to the fiber (its increase indicates myelin damage).

Structural MRI is useful in diagnosing AD; however, it is not sufficient on its own for a definitive diagnosis. Instead, it serves as a supportive tool, particularly for evaluating brain atrophy (volume loss), ventricular enlargement, and cortical thinning. Structural MRI in patients with AD and in its preclinical phase allows for the study of topographic biomarkers of AD. Longitudinal MRI assessments are often used to measure disease progression and evaluate therapeutic efficacy in clinical practice. Gray matter atrophy and volume loss are indicative of neurodegeneration. Atrophy in typical AD begins in the middle temporal lobe and progresses to involve the lateral temporal and parietal cortex [14].

MRI enables the study of overall brain volume as well as the volume of individual brain regions, which can be used for a more accurate assessment in the diagnosis of AD. Indeed, the pattern of affected areas across different cases may help explain the variability in symptom severity. Brain atrophy can be evidenced by regional changes in the early stages. In particular, medial temporal lobe (MTA) atrophy is an important indicator of dementia and is correlated with cognitive decline (measured by the MMSE score). In the early stages, volumetric changes are found in the hippocampus, a region relevant to cognition, and in the entorhinal cortex. This would make the hippocampus a potentially important prodromal marker, as analysis of its volume could contribute to early diagnosis. Frontal atrophy initially occurs in the anterior cingulate, insula, and frontal lobes, associated with impaired language. Posterior and parietal atrophy are characterized by early visual and visuospatial deficits and memory loss. There is also thalamic involvement, including asymmetric atrophy in the ventrolateral and ventromedial nuclei, related to disease severity.

### 1.3 Aim

With this review, we aim to evaluate whether the combined and complementary use of MRS, DTI, and structural MRI could predict the development of AD with high specificity and be used clinically to stratify AD patients, to evaluate cognitive decline (being associated with the MMSE score) and/or to monitor treatment response.

The use of MRS, DTI, and structural MRI allows us to study, respectively, the metabolic, microstructural, and anatomical‐morphological features (especially in brain areas relevant to memory and cognition) that are potentially responsible for the clinical symptoms of AD subjects, associated with the development and progression from MCI and able to reduce symptoms following treatment.

In the present review, we demonstrate that assessing alterations detected through MRI techniques can offer valuable insights for early diagnosis by predicting the conversion and progression from aMCI to AD and monitoring the effectiveness of therapeutic interventions.

## 2 Materials and Methods

To evaluate the efficacy of the combined use of MRS, DTI, and MRI in early diagnosis, we reviewed the recent literature, focusing on clinical studies involving subjects with AD or MCI and normal controls matched for sex and age.

We included clinical trials of therapeutic treatments—pharmacological and non‐pharmacological for AD. For these purposes, we included both cross‐sectional and longitudinal studies.

The research for articles to be selected in this review was performed in the PubMed database using the search terms: “MRS”/“DTI”/“MRI”, “Alzheimer's disease”, “mild cognitive impairment”, “MCI”.

**FIGURE 1:**
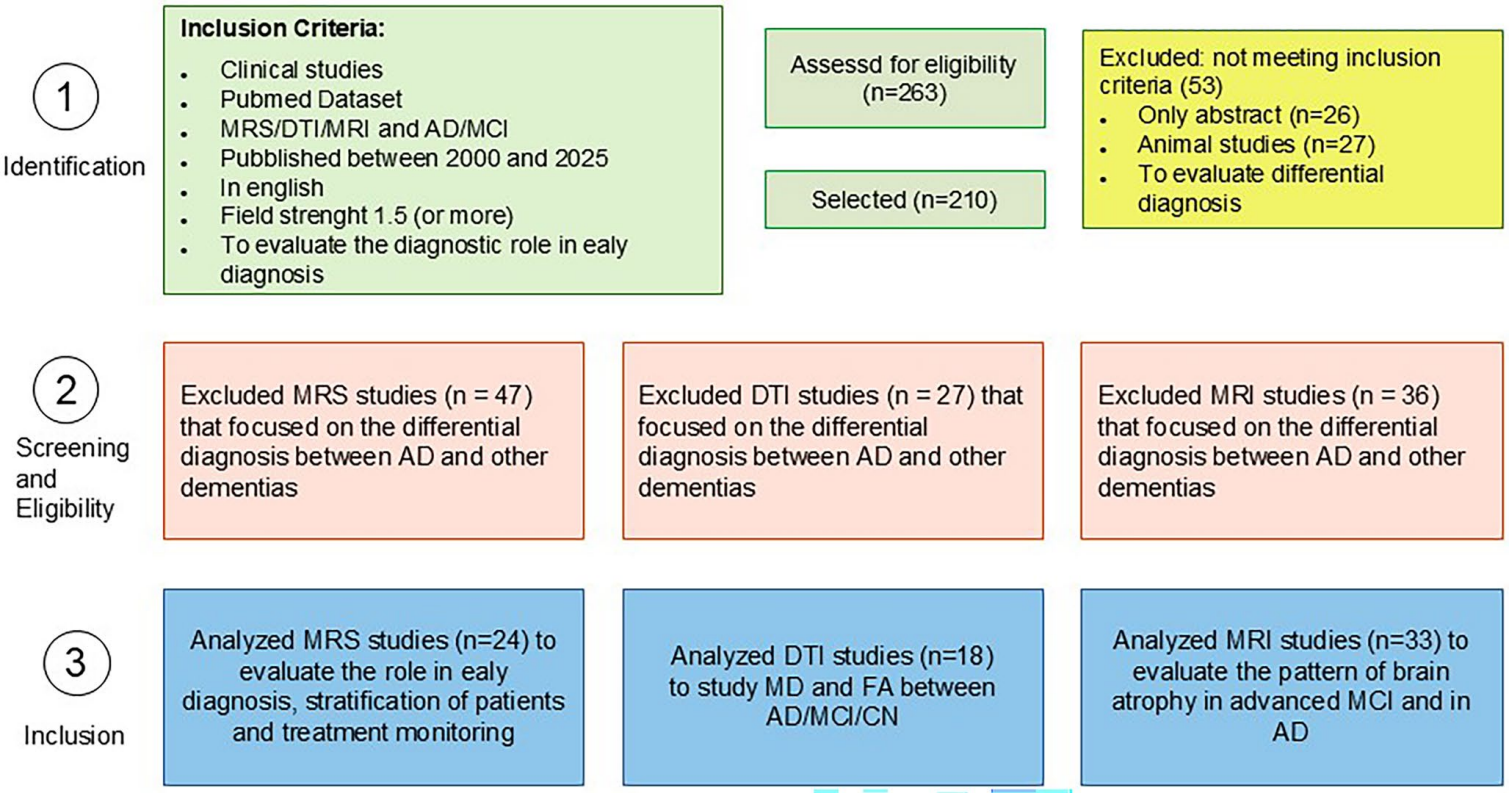
Study selection flow diagram SIGMA. The figure illustrates the inclusion criteria (clinical studies, PubMed dataset, MRS/DTI/MRI in AD/MCI, published between 2000 and 2025, English language, field strength ≥ 1.5 T, and focus on early diagnostic value), the total number of records assessed for eligibility (*n* = 263), and the studies excluded for not meeting the criteria (*n* = 53). The diagram also details the specific exclusions (for differential diagnosis between AD and other dementia) for each imaging technique (MRS, DTI, MRI) and the final number of studies included in the analysis: MRS (*n* = 24), DTI (*n* = 18), and structural MRI (*n* = 33).

Articles published between 2000 and 2025 were searched. The date of 2000 was chosen to include pioneering milestone papers together with the most recent works upgraded to high magnetic fields (up to 7 T). However, these studies, although dated, are evaluated according to the criteria of inclusiveness and high quality. The schematic flowchart is shown in Figure 1.

A manual review of the bibliographic references of relevant articles was also performed to include additional potentially relevant articles. Each article was carefully reviewed and included in our study after an assessment based on the inclusion and exclusion criteria.

Inclusion criteria: articles published in English, in vivo human studies, field strength 1.5 T or higher (3, 4, and 7 T), including participants with AD or MCI and cognitively normal (CN) participants. Single‐voxel MRS techniques for better quality of the spectra and quantitative results.

Data are summarized in Tables 1, 2, 3, 4 which include the conditions under study, number of participants, MRS acquisition methods, and significant key findings. MRS acquisition parameters are highly heterogeneous among the included studies, making their unambiguous and comparable synthesis difficult. For this reason, they have not been reported in detail. DTI and MRI acquisition parameters (Tables 2 and 3), known to be standardized in most studies, were not included as they did not influence the clinical and pathophysiological conclusions discussed. Table 4 summarizes the findings on the role of MRS, DTI, and structural MRI in the evaluation of the efficacy of pharmacological and not pharmacological treatments.

**TABLE 1:** Summary of metabolic findings from MRS studies in AD and MCI.

Reference (Author, Year)	Field strength	TR/TE (ms)	Groups/clinical criteria	Participant's characteristics	Voxel	Diagnostic criteria and key findings	Evidence
Göschel 2024 [15]	7T	6500/9 ms	27 ad Not all AD participants have pathological confirmation via PET or CSF analysis with an increasing concentration of p‐tau181, GFAP, and NfL.	AD: Age = 75 (6); female = 56%; Education [years] = 15 (56%); MMSE 22.68 (4.74)	Sagittal PCC/precuneus region	↑ mI with Increased neuroinflammation and decreased parietal cortical thickness are associated with increases in plasma p‐tau181 and GFAP	Plasma p‐tau181 and GFAP are robust in reflecting 7 T MR‐based changes in AD and disease progression. There were more carriers of (APOE) ε4 in the MCI and AD group compared to SCD and CN groups. Cognitive functions measured by the MMSE, NMM, and executive function lower in the AD.
			30 MCI by neurologists, neuropsychologists, based on neuropsychological test results and clinical CSF.	MCI: Age = 71 (6); female = 27%; Education [years] = 17 (57%); MMSE 27.07 (2.16)			
			35 SCD only plasma p‐tau181 showed a relevant yearly increase. On average, participants from the SCD group showed values like or better than the HC group.	SCD: Age = 69 (7); female = 63%; Education [years] = 11 (31%); MMSE 29.00 (0.97)			
			35 CN	CN: Age = 71 (8); female = 51%; Education [years] = 9 (26%); MMSE 28.97 (1.07)			
Davies‐Jenkins 2024 [16]	7T	3000/14–20 ms TM = 25 ms	13 MCI had a CDR global score of 0.5	MCI: 70 ± 8; female = 23.1%; Years of Education: 15 ± 3; MMSE score: 28 ± 2	ACC, PCC	↓ GABA/Cr and Glu/Cr in the ACC and PCC	Authors examine associations between metabolites, Aβ, and cognitive scores. Reduced GABA/Cr and Glu/Cr in the ACC and PCC would correlate with increased Aβ deposition. Glu + Aβ predicts verbal learning scores better than Aβ‐only models.
			9 LLD (Late‐Life depression) CDR = 0. All LLD patients had a DSM‐IV diagnosis of major depressive disorder with no antidepressant treatment in the past year.	LLD: 70 ± 7; female = 44.4%; Years of Education: 15 ± 2; MMSE score: 29 ± 1			
			13 CN CDR = 0	CN: Age (years) 64 ± 8; female = 53.8%, Years of Education: 16 ± 3; MMSE score: 29 ± 1			
Vints 2024 [17]	3T	2000/30 ms	70 older, stratified by low or high MoCA score for MCI risk. Healthy subjects were stratified by low (< 26/30) or high (≥ 26/30) MoCA scores, indicative of MCI risk. Blood levels of insulin‐like growth factor‐1 (IGF‐1), interleukin‐6 (IL‐6), or kynurenine (KYN) were measured.	Age: 60–85 years; female: 54.2%; MoCa test: 19–30	H subfields: CA1, CA4, subiculum, presubiculum, dentate gyrus	Resistance exercise altered hippocampal subfield volumes. Lower subiculum volume in high‐risk MCI group. Negative correlation between CA1 volume changes and hippocampal tNAA/mIns level changes.	Changes in neurometabolite ratios correlated with volume changes, indicating neuroplasticity effects due to exercise.
Valatkevičienė 2023 [18]	3T	2000/30 ms	22 MCI International Classification of Diseases (ICD‐10) and Petersen criteria (2006).	MCI = 73.1 ± 6.8 years; MoCA 22.5 ± 2.0	LH, R‐DLPFC	↓ NAA/tCr, ↓ NAA/mI, ↑ mI/tCr. Decreased microstructural integrity (lower FA) linked with lower NAA/tCr and NAA/mI and higher mI/tCr.	Decreased tNAA/tCr indicates neuronal density/axonal loss; negative association between mI/tCr and FA suggests increased glial activity disrupting white matter microstructure.
			37 CN	CN = 67.4 ± 5.0 years; MoCA 27.0 ± 1.7			
Kara 2022 [19]	3 T	3000/30 ms	40 CN Petersen et al., 2010	Age: 77.1 (6.7) Female: 48%; Education, years 14.9 (2.6); MMSE: 28.4 (1.1)	PCC	Glu/tCr and NAA/tCr correlated with tau deposition.	This correlation suggest the presence of neuronal and synaptic dysfunction in relation to tau accumulation in PCC.
Wong 2020 [20]	7T	7500/60 ms	9 AD NINCDS/ADRDA criteria	NA	Limbic system	↓ NAA in Left hippocampal and ↓ Glu in posterior cingulate of MCI and AD subjects compared to CN. Differences in DTI metrics indicated volume and white matter loss along the cingulum in AD compared to CN.	Metabolic and microstructural changes were associated with episodic memory performance suggesting that may provide insight into the processes underlining the episodic memory impairment.
			8 MCI Petersen criteria (1999 and 2004)	NA			
			16 CN	NA			
Mitolo 2019 [21]	1.5T	4000/35 ms	23 ad McKhann, 2011 criteria	AD: age = 70.8 ± 9.3; female = 40.0%	PFC, PCC	↓ NAA/mI in PCC (CN > MCI > AD). ↓ parahippocampal and fusiform gyrus volume in converter MCI subgroup versus stable MCI.	NAA/mI in PCC and parahippocampal volume predict conversion from MCI to AD up to 2 years before clinical symptoms.
			38 MCI Petersen, 2004 criteria	MCI: age = 73.9 ± 7.4; years female = 47.3% Progressive = 25.9 (2.7); stable = 26.8 (2.4)			
			18 CN	CN: age = 65.4 ± 9.5; female = 44.44%			
Tumati 2018 [22]	3T	2000/35 ms	28 aMCI (with/without apathy) Petersen 1999 criteria.	Age = 68.5 (4.9); female = 93%; Education: 5.5 (1.1); MMSE 28.6 (1.2); GDS: 7.12 (5.8)	PCC, DACC, R‐DLPFC, R‐TPC	↓ NAA/mI (aMCI without apathy), ↑ Glu, ↑ Gln in DACC (in apathy). ↓ Cho, ↓ mI in TPC (correlated with apathy).	Apathy in aMCI linked to neurometabolic changes indicating altered glial function and membrane integrity.
			20 CN	Age = 67.6 (5.1); female = 100%; Education: 5.7 (0.8); MMSE 28.9 (1.1); GDS: 2.4 (3.6)			
Guo 2016 [23]	3T	1500/35 ms	15 mild AD NIA‐AA criteria (scores ranging from 20 to 24 on MMSE, and a score of 1 on the CDR scale).	AD = age: 72.6 ± 5.12; female: 0%; Education, years: 7.8 ± 1.24; MMSE: 20.5 ± 2.42	L‐ACG, R‐ACG, PCG	In CN, no differences left vs. right in ACG and PCG. In aMCI, mI/Cr in left > right ACG. In AD, mI/Cr and Cho/Cr in left > > right ACG and PCG.	Left–right asymmetry of mI/Cr in ACG and PCG is an important biological marker for mild AD; ACG more susceptible to AD pathology.
			13 aMCI Mayo Clinic Alzheimer's Disease Research Center criteria.	aMCI: age: 73.0 ± 6.14; female: 0%; Education, years: 8.3 ± 1.62; MMSE: 26.1 ± 1.32			
			16 CN	CN = age: 75.1 ± 4.12; female: 0%; Education, years: 8.0 ± 1.61; MMSE: 29.5 ± 0.21			
Zou 2014 [24]	3T	1500/35 ms	20 ad NINCDS‐ADRDA	Age: 64.84 ± 8.82; female: 60%; Education, years: 10.14 ± 3.24; MMSE: 16.21 ± 4.01	PCG, inferior precunei (bilateral)	↓ NAA/Cr, ↑ mI, ↑ Cho/Cr in PCG of AD patients.	MRS of PCG valuable for AD diagnosis.
			20 CN	Age: 64.94 ± 7.93; female: 45%; Education, years: 11.05 ± 4.47; MMSE: 27.35 ± 1.01			
Targosz‐Gajniak 2013 [25]	1.5T	1500/35 ms	41 aMCI Mayo Clinic Group criteria	MCI = age: 72.73 ± 6.88; female: 56.10%; Education, years: 12.29 ± 3.57	PCG, LH, RPL, LPL	29% aMCI progressed to AD in 1 year. ↓ NAA/Cr and NAA/Cho in LH in MCI versus controls. ↑ Cho/Cr, mI/Cr and Glx/Cr in LH. mI/Cr in RPL predicted conversion with 70% sensitivity, 85% specificity.	MRS may predict cognitive decline and conversion to dementia.
			35 CN	NC = age: 71.57 ± 6.27; female: 48.57%; Education, years: 13.66 ± 2.50			
Wang 2012 [26]	3T	1500/35 ms	47 adDSM‐IV criteria and NINCDS/ADRDA criteria	AD: age = 70.9 ± 9.2; Educational (years) = 8.6 ± 5.1; female = 63.8; MMSE = 13.8 ± 5.4; NPI: 9.4 ± 9.6	PCG, H	↓ NAA/Cr in PCC (CN > MCI > AD) ↑ mI/Cr (CN < MCI < AD)	MRS markers in PCG distinguish AD, MCI, and CN.
			32 aMCI Petersen et al., 2001 criteria	aMCI: age = 74.9 ± 7.6; Educational (years) = 10.1 ± 4.0; female = 46.88; MMSE = 23.9 ± 3.8; NPI: 5.2 ± 5.0			
			56 CN	CN: age = 71.4 ± 9.7; Education (years) 10.1 ± 4.0; female = 57.1%; MMSE = 26.5 ± 3.5; NPI: 0.8 ± 3.3			
Watanabe 2010 [27]	1.5T	2000/30 ms	70 adDSM‐IV criteria and NINCDS/ADRDA criteria. Dementia severity in AD was assessed with CDR.	AD = age: 72.1 (7.6); female: 73%; Education, years: 11.4 (0.4); MMSE: 20.8	H, PDWM	NAA_AD_ < NAA_aMCI_ < NAA_CN_ In hippocampus. In the bilateral posterior periventricular and deep white matters (PDWM) and in posterior cingulate gyrus NAA_aMCI_ < NAA_CN_ In the left posterior PDWM tCho ↓	MRS absolute quantification is useful to detect the characteristic patterns of metabolite concentrations in patients with aMCI as compared with AD patients and CN.
			47 aMCI Grundman et al. 2004 criteria	aMCI = age: 71.2 (7.3); female: 48.9%; Education, years: 11.9 (0.5); MMSE: 27.2			
			52 CN	NC = age: 69.4 (6.1); female: 63.5%; Education, years: 10.8 (0.6); MMSE: 29.0			
Kantarci 2008 [28]	1.5T	2000/30 ms	15 CN	CN: Age = 69.48 (6.1); female=: 63.46%; Education, years = 10.8 (0.6); MMSE score: 29.0 (1.4)	L‐PCG, bilateral inferior precunei	↓ NAA/Cr, ↑ mI/Cr associated with higher Braak stage, neuritic plaque score, AD likelihood.	NAA and mI have complementary roles in predicting the AD‐type pathology.
			7 aMCI Petersen 2004 criteria	aMCI: Age = 71.2 (7.3); female = 48.94%; Education, years = 11.9 (0.5); MMSE score: 27.2 (1.8)			
			30 AD DSM‐III‐R criteria for dementia, and NINCDS/ADRDA criteria	AD: Age = 72.1 (7.6); female = 72.86%; Education, years = 11.4 (0.4); MMSE score: 20.8 (3.6)			
Kantarci 2007 [29]	1.5T	2000/30 ms	60 ad DSM‐III‐R criteria for dementia, and NINCDS/ADRDA criteria	AD = age: 77.1 (7.2); female: 55%; Education, years: 14.2 (2.6); MMSE:23.	R‐PCG, L‐PCG, inferior precunei	↓ NAA/Cr, ↑ Cho/Cr and ↑ mI/Cr in aMCI versus CN ↓ Cho/Cr in patients with aMCI who remain stable, but not in patients with aMCI who progress to AD	Cho/Cr decline suggest the presence of a compensatory cholinergic mechanism that may be failing in those aMCI patients who progress to AD. There were fewer APOE ε4 carriers in the control group than all other clinical groups and also fewer APOE ε4 carriers in the aMCI‐stable group than the aMCI‐converter group.
			31 MCI stable DRS, CDR and MMSE score, ApoE genotyping	aMCI‐stable = age: 77.5 (9.2); female: 48%; Education, years: 13.9 (3.4); MMSE:27			
			18 MCI converter DRS, CDR and MMSE score, ApoE genotyping	aMCI‐converter = age: 78.9 (7.1); female: 67%; Education, years: 13.3 (3.5); MMSE:26			
			85 CN	NC = age: 79.1 (7.2); female: 55%; Education, years: 13.9 (2.6); MMSE: 29.			
Metastasio 2006 [30]	1.5T	2000/40 ms	20 stable MCI Peterson's 2001 criteria	aMCI‐stable = age: 73.2 ± 6.1; female: 65%; Education, years: 6.2 ± 3.5; MMSE: 27.2 ± 2.3	Bilateral paratrigonal WM	↓ NAA/Cr in pMCI and sMCI in left hemisphere.	NAA/Cr ratio in paratrigonal WM distinguishes progressive MCI from stable MCI and controls.
			5 Progressive MCI Peterson's 2001 criteria	aMCI‐converter = age: 77.0 ± 2.3; female: 40%; Education, years: 9 ± 5.5; MMSE: 26 ± 1.6			
			29 CN	NC = age: 70.3 ± 3.8; female: 34.5%; Education, years: 6.3 ± 3.4; MMSE: 29.2 ± 0.9			
Godbolt 2006 [31]	1.5T	2000/30 ms	7 presymptomatic mutation carriers (PMCs). Subjects underwent a clinical and neuropsychological assessment (MMSE).	Mean age was 36 years (range 27–50 years) for mutation carriers (four women).	PCC	↓ NAA/Ct and NAA/mI up to 21 years before the expected symptom onset. ↓ NAA/mI is related to proximity of expected age at onset	Metabolic changes are detectable in PMCs years before expected onset of AD.
			6 age‐matched CN	Genetic testing excluding (PS1 and APP mutations). Mean Age was 37 years (range 25–50 years) for control subjects (four women).			
Ackl 2005 [32]	1.5T	2000/35 (WM/GM), or 2000/70 (HPC)	20 AD DSM‐IV, 1994 criteria for dementia and on the criteria of NINCDS‐ADRDA	AD = age: 68.4 ± 10.9; female: 55.6%; Education, years: 9.9 ± 2.1; MMSE: 23.5 ± 4.4	Parietal GM, parietal WM, H	↓ NAA/Cr in hippocampus of MCI and AD. ↑ mI/Cr in PGM, ↓ NAA/Cr in PWM of AD patients	Indicates neuronal loss and gliosis in AD and neuronal loss in MCI.
			20 MCI Mayo Clinic Rochester criteria of Petersen 2001	MCI = age: 66.1 ± 7.8; female: 57.9%; Education, years: 10.1 ± 1.7; MMSE: 29.2 ± 1.1			
			20 CN	NC = age: 63.9 ± 7.7; female: 45.5%; Education, years: 10.5 ± 2.5; MMSE: 29.4 ± 0.8			
Kantarci 2002	1.5T	2000/30 ms	61 NC	NC = age: 80.6 ± 7.2; female:55.7%; Education, years: 14; MMSE: 29	PCG	NAA/Cr can predict conversion from MCI to dementia.	NAA/Cr is a potential biomarker for progression.
			22 ad DSM‐III‐R criteria for dementia, and NINCDS/ADRDA criteria for AD. The severity was rated with CDR score.	AD = age: 79.2 ± 6.1; female: 50%; Education, years: 12; MMSE: 20			
			24 MCI Clinical MCI was definition: CDR of 0.5.	MCI = age: 82.2 ± 5.1; female: 50%; Education, years: 13; MMSE: 28			
Kantarci 2000 [33]	1.5T	2000/30 and 135 ms	21 MCI Clinical MCI was definition: CDR of 0.5	MCI = age: 82.6 ± 5.2; female: 42.9%; Education, years: 12.8 ± 3.3; MMSE: 26.6 ± 2.8	MOL, PCG, L‐STL	↓ NAA/Cr in AD versus MCI and CN in L‐STL and PCG. ↑ mI/Cr in MCI and AD versus controls. ↑Cho/Cr in AD versus MCI and controls.	Initial MRS change in AD progression consist of mI/Cr increase; decrease in NAA/Cr and increase in Cho/Cr develop later.
			21 ad DSM‐III‐R criteria for dementia, and NINCDS/ADRDA criteria for AD. ApoE	AD = age: 79.7 ± 6.3; female: 42.9%; Education, years: 12.9 ± 2.6; MMSE: 18.4 ± 5.9			
			63 CN	NC = age: 80.4 ± 6.9; female: 51.5%; Education, years: 14.0 ± 2.9; MMSE: 28.6 ± 1.3			

*Note*: The columns of the table below represent the following information: The study reference, the magnetic field strength, the MRS relevant acquisition parameters (TR/TE measured in milliseconds), the clinical groups of subjects (categorized into AD, MCI, or CN individuals), the region where the voxel (or ROI) for spectroscopy was placed, the key findings (or metabolite changes) of the study, and the conclusions drawn from the results.

Abbreviations: **Acquisition parameters:** TE = echo time; TR = repetition time. **Brain regions:** GM = Gray Matter; LPL = Left Parietal Lobe; L‐STL = Left Superior Temporal Lobe; MOL = Medial Occipital Lobe; PGM = Parietal Gray Matter; PL = Parietal Lobe; PWM = Parietal White Matter; RPL = Right Parietal Lobe; WM = White Matter. **Groups and diagnostic criteria**: AD = Alzheimer's disease; CN = cognitively normal; Diagnostic and Statistical Manual for Mental Disorders 3rd edition—revised (DSM‐III‐R) criteria for dementia, and 4th edition (DSM‐IV) criteria; MCI = mild cognitive impairment; NIA‐AA = National Institute of Aging and Alzheimer's Association; NINCDS‐ADRDA = National Institute of Neurological and Communicative Disorders and Stroke–Alzheimer's Disease and Related Disorders Association. **Metabolites:** Cho = choline; Cr = creatine; Glu = glutamate; mI = myo‐inositol; NAA = *N*‐acetyl aspartate. **Test:** ADAS‐cog = Alzheimer's Disease Assessment Scale; CDR = Clinical Dementia Rating; GDS = Geriatric Depression Scale; MoCA = Montreal Cognitive Assessment test; NMM = NeuroMET Memory Metric. **Voxel Locations:** ACG = Anterior Cingulate Gyrus; DACC = Dorsal Anterior Cingulate Cortex; H = hippocampus; L‐DLPFC = Left Dorsolateral Prefrontal Cortex; LH = Left Hippocampus; L‐LTL = Left Lateral Temporal Lobe; PCC = Posterior Cingulate Cortex; PCG = Posterior Cingulate Gyrus; PFC = Prefrontal Cortex; R‐DLPFC = Right Dorsolateral Prefrontal Cortex; RH = Right Hippocampus.

**TABLE 2:** Contains articles reporting information on brain microstructure obtained by DTI.

References	Groups/clinical criteria	Participant's characteristics	ROI/diagnostic criteria	Results	Conclusions and efmygdala and nucleividence
Silva‐Rudberg 2023 [34]	33 AD NIA‐AA criteria (CDR score of 0.5 to 1.0, and MMSE score ≤ 26)	Alzheimer's disease (Aβ+) = age: 70.3 (7.7); female: 51.5%; Education, years: 16.4 (2.3); MMSE: 23.3 (3.9)	Gray Matter	Inverse associations between synaptic density and MD in AD patients in regions affected by AD (mainly in the hippocampus)	Link between synaptic loss and gray matter microstructural changes in AD
	17 CN CDR score of 0 and an MMSE score > 26.	Cognitively normal (Aβ−) = age: 72.1 (7.8); female: 47.06%; Education, years: 17.6 (2.0); MMSE: 29.2 (1.1)			
Brueggen 2019 [35]	35 ad NIA‐AA criteria of probable AD	AD = age: 73.5 (6.8); female: 54.29%; Education, years: 13.4 (3.1); MMSE: 23.1 (3.1); GDS score: 2.0 (1.9)	White Matter (corpus callosum, temporal regions)	In corpus callosum and temporal brain regions, ↑ MD: AD > MCI > SCD > CN ↓ FA: AD < MCI < SCD < CN	WM alterations in SCD intermediate between MCI and CN, useful for early detection of progression to AD
	45 MCI NIA‐AA criteria for MCI	MCI = age: 72.3 (5.7); female: 31.11%; Education, years: 14.4 (3.1); MMSE: 28.0 (1.6); GDS score: 1.9 (1.9)			
	98 SCD defined as a persistent self‐perceived cognitive impairment in the absence of objective cognitive impairment	SCD = age: 71.3 (5.9); female: 47.96%; Education, years: 14.6 (3.1); MMSE: 29.3 (0.9); GDS score: 1.8 (1.6)			
	93 CN	CN = age: 68.5 (5.1); female: 59.14%; Education, years: 15.1 (2.6); MMSE: 29.5 (0.8); GDS score: 0.7 (1.5)			
Kantarci 2017 [36]	21 AD low NFT NIA‐AA criteria	21Low NFT Stage = age: 82.7 (5.7); female: 33%; Education, years: 13.9 (3.0); MMSE: 27.3 (2.1); No. of ε4 carriers: 24%; Braak Stage: 2.0 (0.8); CDR: 0.5 (1.4)	White Matter (fornix crus, ventral cingulum, precuneus, entorhinal WM)	AD had significantly ↑ MD and ↓ FA in specified WM tracts; higher MD and lower FA associated with higher Braak NFT stage and clinical severity	NFT pathology is associated with DTI alterations in limbic WM and related to clinical severity
	25 ad high NFT NIA‐AA criteria	25High NFT Stage = age: 75.0 (11.4); female: 32%; Education, years: 14.6 (2.9); MMSE: 16.7 (8.7); No. of ε4 carriers: 42%; Braak Stage: 5.3 (0.7); CDR: 6.6 (5.4)			
Mayo 2017 [37]	34 AD NINCDS/ADRDA had a MMSE score between 20 and 26 and had a CDR of 0.5 (very mild) or 1.0 (mild).	AD = age: 75.8 (7.6); female: 29.41%; Education, years: 15.7 (2.9); MMSE: 23.59 ± 1.74	White Matter (hippocampal cingulate, corpus callosum, internal/external capsule, corona radiata, and so forth)	AD patients had ↓ FA and ↑ MD in multiple WM tracts	White matter microstructure evaluation promising for AD biomarker research
	33 CN	CN = age: 73.0 (6.6); female: 48.48%; Education, years: 16.4 (2.8); MMSE: 29.03 ± 1.26			
Mielke 2012 [38]	23 aMCI analyzed at baseline and at 3, 6, and 12 months; and 2.5 years.	Stable MCI (*n* = 17) Age: 74.5 (5.8); female: 29.4%; education (years): 15.3 (3.1); MMSE: 27.2 (2.0); Memory *Z*‐score: −1.5 (1.0)	Fornix and posterior cingulum DTI measurements and hippocampal volumes	FA, MD, radial diffusivity, and axial diffusivity in the fornix were cross‐sectionally correlated with memory *z* scores.	Both fornix FA and hippocampal volumes were predictive of memory decline.
		Converted to AD (*n* = 6): Age: 78.7 (2.9); female: 33.3%; education (years): 16.3 (3.4); MMSE: 25.0 (1.3); Memory *Z*‐score: −3.2 (0.3)			
Teipel 2012 [39]	137 adNINCDS/ADRCA criteria	AD = age: 72.5 (8.3); female: 57.66%; Education, years: 10.2 (3.3); MMSE: 20.6 (5.3)	White Matter (corpus callosum, temporal lobes, fornix, cingulate, precuneus, prefrontal WM)	AD patients had ↓ FA and ↑ MD	↓ typical pattern of cortical and subcortical microstructural changes in core areas of AD pathology
	143 CN	CN = age: 69.2 (5.9); female: 50.35; Education, years: 13.1 (3.8); MMSE: 28.8 (1.1)			
Liu 2011 [40]	17 ad NINCDS‐ADRDA criteria for a diagnosis of probable	AD = age: 76 (7); female: 64.71%; Education, years: 9 (5); MMSE: 22 ± 5; CDR total: 1.2	White Matter (parahippocampal WM, cingulum, uncinate fasciculus, longitudinal fasciculi, corpus callosum, fornix, brain stem, cerebellar tracts)	↓ FA in AD versus CN in listed tracts; FA values in MCI intermediate between AD and CN	FA values in MCI reflect intermediate microstructural WM alterations between CN and AD
	27 MCI Petersen et al., 2001 and 1999 criteria	MCI = age: 75 (6); female: 44.44%; Education, years: 8 (3); MMSE: 26 ± 2; CDR total: 0.5			
	19 CN	NC = age: (75/6); female: 42.11%; Education, years: 9 (3); MMSE: 28 ±; CDR total: 0.3			
Kantarci 2010 [41]	30 AD NINCDS‐ADRDA criteria for probable AD	AD = age: 74; female: 23%; Education, years: 16; Short Test of Mental Status:24	White Matter (amygdala, ILF, medial temporal, fornix, cingulum)	AD had ↑ MD in medial temporal, temporal, parietal cortices and ↓ FA in fornix, cingulum, ILF	Diffusivity measurements complement structural MRI; useful for characterizing tissue abnormalities in AD
	60 CN	NC = age: 73; female: 17%; Education, years: 14; Short Test of Mental Status: 36			
Sjöbeck 2010 [42]	15 ad NINCDS‐ADRDA criteria of probable AD. The clinical diagnosis of AD was based on clinical/neuropsychiatric and neuropsychological examinations and was further corroborated by structural imaging (CT or MRI), regional cerebral blood flow measurements (SPECT) and tau‐ and b‐amyloid in cerebrospinal fluid.	AD patients = age: 77.5 (1.64); 73.3% female; MMSE score:23.5 (1.09)	White Matter (frontal WM)	↓FA in frontal and occipital WM. Decreased executive functions correlated with greater changes in frontal WM detected by DTI	Structural correlation with executive dysfunctions in AD may relate to changes in deep frontal WM; DTI sensitive to clinically significant WM changes
	15 CN	CN = age 76.5 (1.79); 60% female; MMSE: 29.3 (0.28).			
Cho 2008 [43]	11 MCI Peterson 2004 criteria and CDR 0.5	MCI = age: 72.64 ± 7.33; female: 45.45%; Education, years: 10.91 ± 4.39; MMSE: 24.91 ± 2.43	White Matter (hippocampus, internal capsule, splenium CC, longitudinal fasciculi)	FA ↓ decreased in hippocampus, posterior limb internal capsule, splenium CC, superior and inferior longitudinal fasciculus in MCI versus CN. MD ↑ increased in same areas.	Microstructural changes in corticocortical tracts related to cognition in MCI; FA and MD may be novel biomarkers for neurodegeneration
	11 CN	CN = age: 70.64 ± 2.88; female: 54.55%; Education, years: 9.64 ± 4.61; MMSE: 28.73 ± 0.79			
Kavcic 2008 [44]	14 ad NINCDS‐ADRDA criteria	AD = age74.93 (5.91); Education, years: 14.46 (2.18); MMSE: 24.08 (3.07)	White Matter (corpus callosum, posterior subcortical regions)	Posterior callosal FA correlated with verbal fluency and figural memory impairments; posterior subcortical FA correlated with delayed verbal memory, figural memory, and optic flow perceptual impairments.	Posterior callosal and subcortical WM microstructure relates to cognitive deficits in AD
	18 C	NC = age: 75.39 (7.09); Education, years: 15.67 (2.81); MMSE: 28.94 (1.16)			
Stahl 2007 [45]	15 AD NIA‐AA	AD = age: 68.8 years; female: 53.33; MMSE: 25	White Matter (splenium CC, temporal and parietal WM)	↓ FA and ↓ Relative Anisotropy; ↑ ADC in splenium CC of AD versus MCI; ↑ ADC temporal lobe WM of AD versus MCI and CN; ↑ADC in parietal WM of MCI versus CN	DTI confirms clinical manifestation of AD but less applicable in MCI detection
	16 MCI Petersen, 2001 criteria	MCI = age: 68.9; female: 43.75%; MMSE:27			
	19 CN	CN: age, 63.9 years; female: 57.89%; MMSE: 30			
Firbank 2007 [46]	15 AD NINCDS/ADRDA	AD = age: 76 (7); female: 33.3%; MMSE: 19.1 (4.8); CAMCOG: 67 (11); UPDRS: 5.1 (6.0)	White Matter (parietal lobe, temporal lobe)	↓ FA in parietal lobe of DLB; ↑ ADC in left temporal lobe of AD versus CN	Subtle diffusion imaging changes may reflect disrupted connectivity underlying perfusion changes in DLB and AD
	16 LBD Dementia with LBD cases all met criteria for probable DLB according to the consensus criteria (McKeith et al., 1996).	LDB = age: 76 (7); female: 43.8%; MMSE: 19.1 (4.5); CAMCOG: 66 (15); UPDRS: 30.6 (16.0)			
	15 CN	NC = age: 75 (8); female: 40%; MMSE: 28.3 (2.1); CAMCOG: 97 (4); UPDRS: 0.9 (1.4)			
Zhang 2007 [47]	17 ADNINCDS/ADRDA criteria for probable AD	AD = age: 77.1 ± 8.8; female: 52.94%; Education, years: 14.2 ± 3.6; Short test of mental status: 22.1 ± 4.0; CDR, mean: 1.0 ± 0.4	White Matter (cingulum fibers, splenium of corpus callosum)	↓ FA in cingulum fibers in MCI, more in AD; ↓ FA in splenium CC in AD, not MCI.	Cingulum fiber assessment with DTI may aid early AD diagnosis; combining with hippocampal volume improves accuracy
	17 MCI	MCI = age: 73.1 ± 7.4; female: 47.06%; Education, years: 15.8 ± 2.6; Short test of mental status: 27.9 ± 2.0; CDR, mean: 0.5 ± 0.0			
	18 CN	CN = age: 71.6 ± 9.2; female: 44.44%; Education, years: 15.5 ± 2.7; Short Test of Mental Status: 29.5 ± 0.8; CDR, mean: 0.0 ± 0.0			
Sydykova 2007 [48]	13 AD NINCDS‐ADRDA criteria	AD = age 68.3 (±11.5) years; female: 46.15%; MMSE score: 21.8 (±4.8).	White Matter (anterior e posterior corpus callosum)	FA of anterior CC correlated with GM volume in prefrontal cortex and left parietal lobes in AD; FA of posterior CC correlated with GM volume in bilateral frontal, temporal, right parietal and occipital lobes.	Decline of FA in corpus callosum may relate to neuronal degeneration in corresponding cortical areas
	13 CN	CN: age: 66.7 (±6.4); female: 53.85%; MMSE 29.1 (±0.7)			
Naggara 2006 [49]	Twelve early AD NINCDS‐ ADRDA criteria	12 patients = age = 76.8 ± 4.01 years, range = 67–81 years; female: 58.33; MMSE: 27 ± 2.75	White Matter (splenium CC, frontal, parietal, temporal WM).	↓ FA bilaterally in WM of temporal, frontal lobes, splenium of AD versus controls. ↑ MD in splenium CC and WM of frontal and parietal lobes of AD versus CN.	DTI reveals abnormalities in frontal and temporal WM in early AD; compatible with early temporal‐to‐frontal disconnections
	12 CN	12 CN = mean age = 73.4 ± 4.83 years, range = 66–80 years; MMSE: 30			
Xie 2006 [50]	13 AD NINCDS‐ADRDA criteria	Age: age range 62–82 years; mean age 71.7 years; female = 38.46%; MMSE score ranged from 19 to 24 with mean of 21.1	White Matter (medial temporal lobes, temporal stems, superior longitudinal fasciculi, internal capsules, cerebral peduCNles, middle temporal gyrus, superior parietal lobule, corpus callosum, lateral capsule)	↓ FA in multiple WM regions in AD versus CN.	WM degeneration pattern differs from GM and may independently contribute to AD progression
	16 CN	Age: 61 to 79 years; mean age 71 years; female: 37.5%			

Abbreviations: ADC = Apparent Diffusion Coefficient; CC = Corpus Callosum; DAT = Dementia of the Alzheimer Type; FA = Fractional Anisotropy; GM = Gray Matter; ILF = Inferior Longitudinal Fasciculus; MD = Mean Diffusivity; NFT = Neurofibrillary Tangles; RA = Relative Anisotropy; ROI = region of interest; SCD = Subjective Cognitive Decline; WM = White Matter.

**TABLE 3:** Summary of structural alterations derived from MRI studies in AD and MCI.

Author, Year	Groups/clinical criteria	Participant's characteristics	ROI	Findings	Conclusion
Vilor‐Tejedor 2025 [51]	214 ad, from ADNI dataset/APOE and PET	AD mean age = 74.6 (7.8); female: 46.3%.	Hippocampus and subregions (subiculum, presubiculum e parasubiculum)	AD genetic risk (especially the APOE region) accelerates a reduction in hippocampal volume, particularly in individuals with MCI	AD genetic factors—especially the APOE region—exert their main influence before or during the transition to MCI, when the hippocampus is particularly vulnerable
	474 MCI	MCI mean age = 73.8 (7.1); female: 39.0%			
	363 CN	CN mean age = 74.3 (6); female 52.1%			
Langella 2024 [52]	27 PSEN1 mutation carriers	PSEN1 mutation carriers: age: 35.93 (8.18); female: 70.37%; Education, years: 10.19 (3.26); MMSE: 28.74 (1.87)	Hippocampus	Smaller hippocampal volume associated with depressive symptoms	Hippocampal neurodegeneration can be linked to depressive symptoms in autosomal dominant AD
	26 non‐carriers family members	Non‐carriers family members: Age: 37.04 (6.48); female: 88.46% Education, years: 10.58 (4.67); MMSE: 29.69 (0.55)			
Steinbart 2023 [53]	33 AD ADNI clinical data repository	AD = mean age in years 74.0 (8.1); female: 66.67%	Piriform Cortex (PC)	PC volumes lower bilaterally in MCI and AD. In patients with MCI and AD, PC volumes were ↓ than those of CN bilaterally.	Early atrophy of PC (involved in olfaction and memory) at the stage of MCI may represent a new biomarker.
	71 MCI	MCI = mean age = 75.0 ± 8.1; female: 36.6%			
	47 CN	CN = MEAN AGE: 75.1 ± 3.9; female: 61.70%			
Mathew 2023 [54]	28 CN	CN = age: 70.5 (5.8); female: 78.6%; MoCA Total Score: 26.4 (2.1); CDR‐Sum of Boxes: 0.04 (0.1)	Retinal nerve fiber layer (RNFL)	RNFL thickness in both eyes was positively associated with brain volumes in subjects with cognitive decline.	Mean RNFL thickness was correlated with brain volumes, representing a useful non‐invasive marker for dementia diagnosis.
	26 SCD	SCD = age: 71.0 (5.6); female: 57.7%; MoCA Total Score: 25.4 (3.8); CDR‐Sum of Boxes: 0.2 (0.4)			
	17 MCI	MCI = age: 73.8 (7.5); female: 41.2%; MoCA Total Score: 20.6 (4.0); CDR‐Sum of Boxes: 1.1 (1.2) CDR‐Sum of Boxes: 3.5 (1.4)			
	4 AD ADNI clinical data repository	AD = age: 68.6 (12.0); female: 25.0%; MoCA Total Score: 15.5 (7.0)			
Carnemolla 2022 [55]	50 ad ADNI clinical data repository	AD = age: 65.7 (7.7); female: 42%; Education, years:12.2 (3.0)	Olfactory bulb (OB)	At first access, in AD and FTD, OB volumes were similar to CN. Olfactory bulb volume atrophied (10%–25% volume reduction) significantly later (12 months) in the disease process.	The volume of the olfactory bulb decreases with the progression of AD and FTD.
	119 FTD	FTD = age: 64.1 (7.5); female: 41.8%; Education, years: 11.9 (2.9)			
	55 CN	CN = age: 65.1 (7.7); female: 63.6%; Education, years: 13.4 (2.6)			
Bernstein 2021 [56]	89 ad, 212 early MCI, 114 late MCI, 125 CN ADNI database	AD = age: 74.06 (7.74); female: 47%; Education, years: 16.08 (2.52); MMSE: 23.01 (2.20)	Thalamic nuclei	The volumes of anteroventral, mediodorsal, pulvinar, medial geniculate and centromedian nuclei are reduced in subjects with late MCI and AD compared to CN.	Atrophy of thalamic nuclei is associated with cognitive alterations in AD and memory decline.
		LMCI = age: 71.81 (7.93); female: 53%; Education, years: 16.61 (2.50); MMSE: 27.67 (1.81)			
		EMCI = age: 70.60 (7.16); female: 49%; Education, years: 16.01 (2.69); MMSE: 28.44 (1.55)			
		CN = age: 73.42 (6.25); female: 55%; Education, years: 16.62 (2.47); MMSE:29.10 (1.16)			
van de Mortel 2021 [57]	239 ad NINCDS/ADRDA criteria	AD = age: 75.4 ± 7.65; female: 51%; Education, years: 15.1 ± 3.04; MMSE: 23.1 ± 2.02 ADNI clinical data repository	GM: thalamus, hippocampus, parahippocampal gyrus, amygdala, and so forth.	Early gray matter loss in hippocampus, thalamus, and amygdala in MCI and AD. Thalamic volume loss in early MCI and pulvinar reduction in AD. Conversion from MCI to dementia associated with reduced GM volume in several brain areas: thalamus, amygdala, bilateral hippocampus, parahippocampal gyri, medial/inferior temporal gyri, plus angular gyrus, precuneus, insula, midfrontal, and midoccipital cortex.	The thalamic volume reduction is one of the first signs of cognitive decline and may therefore be a key player in dementia besides hippocampal regions, which did not show any alterations in the earliest stages of cognitive impairment. This study proposes an additional role of the (posterior) thalamus in the cognitive deterioration that characterizes AD.
	501 LMCI	LMCI = age: 74.0 ± 6.43; female: 4.9%; Education, years: 15.9 ± 2.95; MMSE: 27.1 ± 1.80			
	295 EMCI	EMCI = age: 71.0 ± 7.47; female: 43.0%; Education, years: 15.9 ± 2.64; MMSE: 28.3 ± 1.55			
	351 CN	CN = age: 75.0 ± 5.79; female: 53.8%; Education, years: 16.3 ± 2.70; MMSE: 29.1 ± 1.13			
Iritani 2021 [58]	48 ad DSM V	Age 79.5 (6.8) years (range 65–93 years). Female: 65.2%; MMSE = 23	Olfactory bulb	Significant shrinkage of the frontal lobe in participants with sarcopenia, and shrinkage of the medial temporal areas and global brain in participants with Kihon Checklist frailty/dependence.	The olfactory‐cognitive index might be a useful tool to distinguish involvement of frontal lobe shrinkage, in older adults in progression to AD
	23 MCI	Age = 81.3 (8.1); 62.5%; MMSE: 25.6 (0.9)			
	64 CN	Age = 77.2 (5.9); female: 69.6%; MMSE: 29.5 (0.8)			
Dutt 2020 [59]	814 CN		Brainstem (midbrain, LC, pons, entire brainstem)	AD/MCI cases showed smaller volumes of midbrain, locus coeruleus, pons and entire brainstem compared to healthy subject. Reduced volume of midbrain and LC associated with poorer performance in tests of attention and executive function in AD and in aMCI individuals.	Confirms progressive atrophy in the brainstem in AD. Brainstem degeneration linked to attention and executive deficits in the prodromal stage of Alzheimer's disease.
	542 aMCI ADNI diagnostic criteria	Age: 55–90 years			
Yoo, 2020 [60]	42 ADCI NINCDS/ADRDA The presence of ADCI was identified using PET and Petersen criteria. The presence of ADCI was identified using (18F‐FBB) PET.	Pure ADCI = age: 72.1 (8.3); female: 66.7%; Education, years: 10.6 (4.8); MMSE: 22.0 (3.8)	Amygdala, hippocampus, thalamus, pallidum, putamen, caudate	Subcortical atrophy pattern differs among types—ADCI and mixed disease groups had a reduction in hippocampal and amygdala volumes compared to CN and LBCI groups. LBCI and mixed disease groups had a reduction in putamen volume compared to ADCI and CN and a reduction pallidum volume than pure ADCI and mixed disease groups.	ADCI and LBCI were independently associated with subcortical atrophy in a disease‐specific manner: in the hippocampus and amygdala and in the hippocampus and putamen, respectively.
	30 LBCI The presence of LBCI was supported by abnormalities in (18F‐FP‐CIT) PET scans.	Pure LBCI = age: 75.6 (8.6); female: 53.3%; Education, years: 10.1 (5.0); MMSE: 23.3 (3.5)			
	58 mixed	Mixed disease: age: 73.7 (7.8); female: 52.5%; Education, years: 10.5 (5.5); MMSE: 20.4 (4.4)			
	29 CN	CN = age: 71.7 (4.9); female: 58.6%; Education, years: 10.5 (5.0); MMSE: 28.4 (1.3)			
Boublay, 2020 [61]	53 ad CDR criteria	AD = age: 78 (5); female: 56.6%; MMSE: 24 (2); Depression item score: 0.22; CDR: 0.7	Cortical and cerebellar areas	Involvement of more structures (in the frontal, temporal, parietal, occipital, subcortical regions and cerebellum) were associated with an increased risk of developing BPSD, predicting the evolution of NPI scores	The volume of the frontal lobe (with frontal gyri, anterior cingulate cortex, and orbital gyri) is the strongest predictor for NPI scores and suggesting their involvement in cognition.
	40CN	CN: age: 77 (4); female: 65%; MMSE: 30 (0); Depression item score: 0; CDR: 0			
Vuoksimaa 2020 [62]	230 CN	NC = age:76.12 (5.02); Education, years: 16.03 2.85; AVLT1 5.17 (1.66)	Medial temporal lobe (hippocampus, entorhinal cortex)	AVLT−group showed more medial temporal atrophy; smaller hippocampal volumes and thinner entorhinal cortex than AVLT+ and CN.	AVLT−group had a higher risk of progression to AD than the AVLT+.
	394 MCI (121 AVLT+, 273 AVLT−) Petersen 2010 criteria	MCI = age: 74.92 (7.44); Education, years: 15.67 3.04; AVLT1 4.19 (1.53)			
Tabatabaei‐Jafari, 2017 [63]	191 ad NINCDS/ADRDA criteria	CN = age: 75.87 (5.02); female: 48%; Education, years: 16.07 (2.86); MMSE: 29.11 (0.99)	Cerebellum	Cerebellar volume reduction of 2.5% in AD; no differences between CN–MCI and MCI–AD.	Cerebellar atrophy occurs in advanced AD and is secondary to cerebral involvement.
	398 MCI MMSE score higher than 24, CDR of 0.5	MCI = age: 74.74 (7.39); female: 35%; Education, years: 15.64 (3.03); MMSE: 27.03 (1.78)			
	229 CN MMSE score higher than 24, a CDR of 0.	AD = age: 75.27 (7.0); female: 47%; Education, years: 14.70 (3.15); MMSE: 23.31 (2.04)			
Agüera‐Ortiz 2017 [64]	37 moderate to severe AD	AD = age is 82.7 (5.8); female:78.4%; MMSE: 15.3 (9.0)	Corpus callosum and internal capsule	Bilateral damage to the corpus callosum and internal capsule was associated with the severity of apathy. A smaller and more anteriorly located region of the right internal capsule and corpus callosum was associated with greater emotional blunting. Ischemic damage to the right periventricular frontal region was associated with greater thinking deficits.	Ischemic and structural damage in CC and anterior internal capsule is associated with emotional dullness, apathy, and disorganized thought
Makovac 2016 [65]	Probable AD with BPSD NINCDS‐ADRDA criteria	AD = age: 71.9 (±7.2); female: 74%; Education, years: 9.6 (±4.42); MMSE: 19.0 (±3.9); CDR score 1.5 (±0.7)	WM (Corpus callosum‐CC) and GM	WM alterations in CC and GM atrophy in BPSD.	Supports the hypothesis that specific patterns of WM and GM neurodegeneration may be potential diagnostic and prognostic markers in AD
Torso (2015) [66]	31 aMCI MMSE score had to fall above the cut‐off for normality (> 23.8), CDR score = 0.5.	a‐MCI = Age: 71.3 (8.1); female: 45.2%; MMSE: 26.0 (1.6)	Anterior thalamic radiations (ATR)	ATR damage linked to apathy in aMCI patients; GM atrophy not associated with BPSD.	Voxel based lesion symptom mapping (VLSM) showed association between ATR lesions and apathy severity.
	26 CN	CN = Age: 67.5 (7.0); female: 42.3%; MMSE: 28.6 (1.4)			
Brueggen 2019 [67]	35 AD DELCODE dataset NIA‐AA criteria	AD = age: 73.5 (6.8); female: 54.3%; Education, years: 13.4 (3.1); MMSE: 23.1 (3.1); GDS score: 2.0 (1.9)	Hippocampus, basal forebrain, other AD‐related areas	Reduced volume of the hippocampus, basal forebrain, and other AD‐related regions predicted increased risk of conversion from MCI to AD.	Reduced right hippocampal volume is the most significant predictor of conversion to AD
	45 MCI NIA‐AA criteria	MCI = age: 72.3 (5.7); female: 31.1%; Education, years: 14.4 (3.1); MMSE: 28.0 (1.6); GDS score: 1.9 (1.9)			
	98 SCD	SCD = age: 71.3 (5.9); female: 48%; Education, years: 14.6 (3.1); MMSE: 29.3 (0.9); GDS score: 1.8 (1.6)			
	93 NC	CN = age: 68.5 (5.1); female: 59.1%; Education, years: 15.1 (2.6); MMSE: 29.5 (0.8); GDS score: 0.7 (1.5)			
Elcombe 2015 [68]	218 elderly at risk of cognitive decline	mean age = 67.3 years, MMSE = 28.6	Hippocampus	Hippocampal volume reduction is associated with increased risk of cognitive decline.	Hippocampal atrophy may serve as an early biomarker for cognitive decline in elderly populations.
Peng 2015 [69]	38 ad NINCDS/ADRDA criteria	AD = age: 75.5 (5.1); female: 44.7%; Education, years: 9.8 (2.2); MMSE: 21.5 (2.2)	Hippocampus (baseline +2 follow‐ups)	AD patients had smaller hippocampal volumes than aMCI and CN	Strong correlation between hippocampal volume and cognitive performance (MMSE, AVLT, ADL, BNT)
	22 aMCI Petersen 2001 criteria	MCI = age: 74.2 (4.2); female:54.5%; Education, years: 9.6 (2.3); MMSE: 27.7 (1.1)			
	20 CN	CN = age: 73.7 (4.5); female: 45.0%; Education, years: 10.1 (1.7); MMSE: 28.9 (0.8)			
Lee 2015 [70]	50 AD NINCDS‐ADRDA criteria CDR ≥ 1.	AD = age:72.1 ± 3.8; female: 52%; Education, years: 9.4 ± 3.1; MMSE: 21.4 ± 3.3; CDR: 1.8 ± 1.2	Brainstem	Reduction of total brainstem volume; significant deformation in upper posterior brainstem (midbrain) in AD patients.	Midbrain structural changes could lead to brainstem dysfunction, a region implicated in cognitive and behavioral symptoms (memory impairment, sleep, and emotional disturbances in AD) and may contribute to cognitive and behavioral symptoms in AD.
	50 CN	CN = age: 71.2 ± 4.3; female: 54%; Education, years: 9.0 ± 4.2; MMSE: 28.4 ± 1.5; CDR: 0			
Vasavada 2015 [71]	15 AD NINCDS‐ADRDA criteria	AD = age: 71.9 ± 11.9; female: 66.7%; Education, years: 14.3 ± 3; MMSE: 18.9 ± 5.4	Piriform/olfactory cortex (POC), hippocampus	Prominent atrophy in POC and hippocampus in MCI and AD correlated with behavior.	Olfactory deficits in MCI and AD may precede cognitive symptoms and memory deficits. Olfactory deficits are prevalent in AD and MCI patients.
	21 MCI Peterson 1999 criteria	MCI = age: 73.2 ± 9.0; female: 52.4%; Education, years: 14.6 ± 2.9; MMSE: 26.5 ± 1.9			
	27 CN	CN = age: 69.5 ± 10.4; female: 55.6%; Education, years: 16.0 ± 1.7; MMSE: 28.5 ± 1.5			
Kilimann 2014 [72]	134 ad NINCDS‐ADRDA criteria	AD: age: 72.5 (8.3); female: 56.7%; Years of education: 10.2 (3.4); MMSE: 20.7 (5.4)	Basal forebrain cholinergic system (BFCS)	AD: volume reduction in all BFCS subregions; MCI: volume reduction in the posterior NbM, but preserved volumes in the anteromedial regions.	BFCS volumetry could serve as a biomarker for early AD.
	41 MCI	MCI: age: 69.2 (5.9); female: 50.0%; Years of education: 13.1 (3.8); MMSE: 28.8 (1.1)			
	148 CN	CN: age: 70.5 (6.4); female: 41.5%; Years of education: 12.1 (2.9); MMSE: 25.7 (4.4)			
Apostolova 2012 [73]	43 ad NINCDS‐ADRDA	AD = age: 75.7 (7.6); Education, years: 14.6 (2.7); MMSE: 22.2 (4.9)	Hippocampus and ventricles	Hippocampal atrophy and ventricular enlargement were observed in the AD compared to CN. aMCI patients showed intermediate levels of hippocampal atrophy and ventricular dilation.	Normal aging is also associated with hippocampal atrophy and ventricular dilation. Unlike in AD, these changes are modest and their rate of progression over time is relatively slow.
	33 MCI Petersen 2001 criteria	MCI = age: 73.1 (6.0); Education, years: 16.3 (2.6); MMSE: 27.8 (2.3)			
	6 CN	CN = age: 66.4 (7.8); Education, years: 17.2 (2.6); MMSE: 29.5 (0.6)			
Cavedo 2011 [74]	19 ad	AD = mean age: 76 (6); MMSE:13 (4)	Amygdala and nuclei	Reduction of bilateral amygdalar volume in AD group; local tissue loss was mapped in the medial, central nuclei, lateral nucleus (La) and basolateral ventral medial nucleus (BLVM).	Amygdalar subregions connected to hippocampus (BLVM, La) and olfactory/cholinergic systems (respectively, medial nucleus and central nucleus) are affected in AD.
	19 CN	CN = age: 74 (5), MMSE: 29 (1)			
Li, 2012 [75]	227 ad	NA	G (hippocampus, entorhinal cortex, medium temporal gyrus—MTG)	Hippocampus and entorhinal cortex are affected in early AD. MTG is involved in late AD.	Greater differences between AD and CN are observed in MTG, suggesting its key role in intermediate stages of the disease
	215 CN	NA			
Carmichael 2010 [76]	804 ad (followed 1 year)	Varying degrees of cognitive impairment who were diverse across the number of relevant demographic variables	WM	Higher baseline WM hyperintensities volume was associated with greater subsequent 1‐year increase in ADAS‐Cog and decrease in MMSE scores. Greater WM hyperintensities volume at follow‐up was associated with greater ADAS‐Cog and lower MMSE scores at follow‐up.	White matter hyperintensity volume predicts 1‐year cognitive decline
Thomann 2009 [77]	21 early AD NINCDS‐ADRDA criteria	AD = age: 71.76 ± 4.94; female: 66.7%; Education, years: 9.81 ± 1.94; MMSE: 22.10 ± 1.87	Olfactory bulb tract (OBT)	Reduction of right, left and middle OBT volume in early AD; OBT volume correlated with MMSE.	OBT atrophy is present already in the early stages of AD and progresses with disease severity.
	21 CN	CN = age: 70.38 ± 7.14; female: 61.9%; Education, years: 9.67 ± 1.74; MMSE: 29.0 ± 0.81			
Risacher 2009 [78]	148 ad ADNI clinical data repository	AD = age: 75.4 (0.6); Education, years: 14.8 (0.2); MMSE: 23.5 (0.1)	Global and hippocampal gray matter (GM) density, hippocampus and amygdala volumes, cortical thickness of the entorhinal cortex	↓ Bilateral hippocampus and amygdala volume, entorhinal cortex thinner; Greater involvement of left‐sided regions. MCI‐Converters and AD showed no significant differences in any MTL measures (hippocampus, amygdala, or entorhinal cortex thickness).	MCI‐Converters show more MTL atrophy than MCI‐Stable.
	339 MCI	*n* = 277 MCI: MCI‐progressive = age: 74.3 (0.9); Education, years: 15.2 (0.4); MMSE: 26.7 (0.2) MCI‐stable = age: 75.1 (0.4); Education, years: 15.8 (0.2); MMSE: 27.1 (0.1)			
	206 CN	CN = age: 76.0 (0.5); Education, years: 16.1 (0.2); MMSE: 29.1 (0.1)			
De Jong 2008 [79]	69 ad NINCDS‐ADRDA criteria	AD = age: 77 (7.4); Education, years: 9.8 (4.0); MMSE: 18 (4.7)	Nucleus accumbens, amygdala, caudate nucleus, hippocampus, pallidum, putamen and thalamus	Reduction of putamen, thalamus and hippocampus volumes in AD. These volume reductions were associated with cognitive decline.	Degenerative processes in the putamen, thalamus and hippocampus, may contribute to cognitive decline in AD. There are an association between hippocampal atrophy and cognitive decline in AD
	70 MCI	MCI = age: 66 (12) Education, years: 12 (3.7); MMSE: 27 (2.3)			
Whitwell 2008 [80]	85 MCI groups (aMCI‐Stable versus aMCI‐Progressors) DSM IV and NINCDS‐ADRDA criteria	Braak NFT stage III‐VI (III = 16; IV = 11; V = 31; VI = 27)	GM patterns: temporal lobes, neocortex, cingulate	GM loss in medial/lower temporal lobes, temporoparietal neocortex, cingulate, and frontal lobes in aMCI‐P compared to control group. The aMCI‐S group showed no regions of gray matter loss compared to controls. The aMCI‐P group showed greater loss in the medial and inferior temporal lobes, temporoparietal neocortex, posterior cingulate, precuneus, anterior cingulate, and frontal lobes compared to the aMCI‐S group.	aMCI‐P shows more widespread GM loss than aMCI‐S
Duarte 2006 [81]	14 AD NINCDS‐ADRDA criteria	AD = age: 74.6 (11.4); female: 35.7%; Education, years: 14.9 (4.7); MMSE: 21.3	Frontal, temporal, parietal regions	AD demonstrated substantial loss in temporal, parietal and frontal regions while MCI exhibited moderate volume loss in temporal and frontal regions.	MCI shows intermediate atrophy between normal aging and AD.
	32 MCI MCI patients had cognitive complaints and objective impairments in either episodic memory and/or executive functioning.	MCI = age: 74.1 (6.0); female: 34.4%; Education, years: 15.3 (3.5); MMSE: 28.0			
	14 CN	CN = age: 69.5 (7.4); female: 57.1%; Education, years: 16.4 (2.0); MMSE: 29.5			
Pennanen 2004 [82]	48 AD DSM‐IV and NINCDS‐ADRDA criteria	AD = age: 71.1 (8.1); female: 52.1%; Education, years: 6.8 (3.4); MMSE: 21.4 (3.5)	ERC, hippocampus	Volumes of the hippocampus and ERC were significantly reduced in the following order: control > MCI > AD.	ERC atrophy precedes hippocampal atrophy in AD.
	65 MCI Mayo Clinic criteria	MCI = age: 72.8 (4.5); female: 66.15%; Education, years: 6.7 (1.6); MMSE: 24.0 (2.5)			
	59 CN	CN: age: 72.7 (4.3); female: 62.7%; Education, years: 8.1 (3.2); MMSE: 27.3 (1.8)			

Abbreviations: AD = Alzheimer's Disease; ADCI = Alzheimer Disease related Cognitive Impairment; ADL = Activities of Daily Living; ADNI = Alzheimer's Disease Neuroimaging Initiative database; aMCI = Amnestic Mild Cognitive Impairment (MCI with predominant memory deficits); AVLT = Auditory Verbal Learning Test; BFCS = Basal Forebrain Cholinergic System; BNT = Boston Naming Test; BPSD = Behavioral and Psychological Symptoms of Dementia; bvFTD = Behavioral Variant Frontotemporal Dementia; CC = Corpus Callosum; CN = Cognitively Normal; ERC = Entorhinal Cortex; FTD = Frontotemporal Dementia; GM = Gray Matter; LBCI = Lewi Body related Cognitive Impairment; LC = Locus Coeruleus; MCI = Mild Cognitive Impairment; NA = not available; OB = Olfactory Bulb; PC = Piriform Cortex; PNFA = Progressive Non‐Fluent Aphasia; RNFL = Retinal Nerve Fiber Layer; ROI = Region of Interest (anatomical/study region); rTMS = Repetitive Transcranial Magnetic Stimulation; SD = Semantic Dementia; VLSM = Voxel‐based Lesion Symptom Mapping; WM = White Matter.

**TABLE 4:** Summary of the findings on the role of MRS, DTI and structural MRI in the evaluation of the efficacy of pharmacological and not pharmacological treatments.

Reference (Author, Year)	Field strength	TR/TE (ms)	Groups of treatment	Participant's characteristics/clinical criteria	Voxel/region of interest	Key findings	Evidence
Rash 2025 [83]	3T	DTI and MRI: 1800/2.5 ms; TI = 900 ms	AD (*n* = 11) treated with larometrocel (100 million cells; four monthly doses)	AD = age: 74.1 (6.65); female: 55.1%; MMSE‐2 at baseline: 21.2 (2.05); CDR: 4.23 (1.62); ADAS‐Cog‐13: 29.09 (9.36); MoCA total score: 18.1 (4.83); ApoE4 status: 71.4%; AD treatment (galantamine, memantine and combined): 63.3%. PET and fluid biomarkers	MRI: whole brain, DTI: MD and FA in the cingulate cortex.	Laromestrocel improved clinical assessments at 39 weeks compared to placebo, as measured by a composite AD score. Laromestrocel slowed the decline of whole brain volume compared to placebo and reduced neuroinflammation as measured by DTI	This study provide indications of efficacy in combating decline of brain volume and potentially cognitive function
			AD (*n* = 13) treated with larometrocel (25 million cells; four monthly doses)				
			AD (*n* = 13) treated with laromestrocel (25 million cells; 1 dose followed by placebo)				
			AD (*n* = 12) treated with placebo (four monthly doses)				
Abushakra 2025 [84]	n.a.	DTI and MRI: TR/TE = *n*.a.	MCI and early AD treated with valiltramiprosate (265 mg twice/day) *n* = 163	AD subject carrying APOE4/4 genotype. Diagnosis is based on: Imaging MRI biomarkers at baseline and weeks 26, 52, and 78—fluid biomarkers: Aβ42, Aβ40, p‐tau181, p‐tau217, GFAP, and NfL. In addition, serum levels of sTIE2. Neuropsychiatric tests: MMSE, CDR and ADAS‐Cog13.	MRI Region of interest: bilateral hippocampal volume, Cortical thickness, whole brain volume and ventricular volume. DTI on white and gray matter	The APOE4/4 early AD population did not show significant clinical efficacy at 78 weeks but showed significant brain atrophy slowing. Prespecified analyses at the MCI stage showed significant slowing of clinical decline and hippocampal atrophy slowing.	
			MCI and early AD treated with placebo *n* = 162				
Zhang 2019 [85]	3T	MRS: TR/TE = 1500/35 ms	15 AD real rTMS: NINCDS‐ADRDA	Age: 69.00 ± 8.19; female = 80.0%; Education (y) = 12.40 ± 2.06; MMSE score = 20.53 ± 4.17; NPI = 10.20 ± 4.65	L‐DLPFC, L‐LTL	↑ NAA/Cr in L‐DLPFC (treated group). Trend to increase in NAA/Cr in L‐LTL (real group)	Increase in NAA/Cr in L‐DLPFC correlated with cognitive improvement; L‐DLPFC is promising rTMS target in AD.
			15 AD sham rTMS NINCDS‐ADRDA	Age = 69.00 ± 8.19 Education (y) = 11.85 ± 2.38; MMSE = 19.83 ± 5.10; NPI = 8.80 ± 4.21			
Penner 2010 [86]	4T	MRS: TR/TE = 3200/46 ms	10 ad NINCDS/ADRDA criteria for probable AD	Baseline = age: 81.5 ± 6.0; female: 70%; Education, years: 12.9 ± 3.8; MMSE: 25.9 ± 2.0, ADAS‐cog: 15.2 ± 4.2 4 months = age81.9 ± 6.0; MMSE: 24.7 ± 1.9; ADAS‐cog: 15.1 ± 6.5	RH	↑ Glu, ↑ Glu/Cr, and ↑ Glu/NAA after 4 months galantamine in AD versus CN.	Galantamine may improve glutamatergic transmission.
Modrego 2006 [87]	1.5T	MRS: TR/TE = 2500/30	24 ad DSM‐IV criteria	Baseline ADAS‐cog = 25.04 (6.1). ADAS‐non‐cog: 16.3 (4.1) After treatment ADS‐cog = 24.04 (6.5). ADAS‐non‐cog: 15.9 (4.4)	Frontal, parietal, occipital cortices.	Rivastigmine reversed ↓ NAA/Cr in frontal cortex.	Cholinergic treatment may restore neuronal function (↑ NAA/Cr).
Krishnan 2003 [88]	1.5T	MRS: TR/TE = 1200/35 ms	33 ad DSM‐IV‐TR and NINCDS‐ADRDA	Patients Receiving donezepil: age = 74.4 (7); female = 74%; MMSE = 19.5	Subcortical GM, periventricular, cortical, WM at third ventricle	↑ NAA in donepezil‐treated patients versus placebo.	Donepezil may improve neuronal metabolism (↑ NAA).
			34 AD with placeboDSM‐IV‐TR and NINCDS‐ADRDA	Patients Receiving Placebo: Age = 72.4 (10); female = 70%; MMSE = 19.0			

*Note*: The columns of the table below represent the following information: The study reference, the magnetic field strength, the MRI, DTI and MRS relevant acquisition parameters (TR/TE measured in milliseconds), the clinical groups of subjects (categorized into AD, MCI, or CN individuals), the region where the voxel (or ROI) was placed, the key findings (or metabolite changes) of the study, and the conclusions drawn from the results.

Abbreviations: **Acquisition parameters:** TE = echo time; TR = repetition time. **Brain regions:** GM = Grey Matter; LPL = Left Parietal Lobe; L‐STL = Left Superior Temporal Lobe; MOL = Medial Occipital Lobe; PGM = Parietal Grey Matter; PL = Parietal Lobe; PWM = Parietal White Matter; RPL = Right Parietal Lobe; WM = White Matter. **Diagnostic criteria:** NIA‐AA = National Institute of Aging and Alzheimer's Association; NINCDS‐ADRDA = National Institute of Neurological and Communicative Disorders and Stroke–Alzheimer's Disease and Related Disorders Association; Diagnostic and Statistical Manual for Mental Disorders 3rd edition—revised (DSM‐III‐R) criteria for dementia, and 4th edition (DSM‐IV) criteria. **Groups of treatment**: AD = Alzheimer's disease; CN = cognitively normal; MCI = mild cognitive impairment. **Metabolites:** Cho = choline; Cr = creatine; Glu = glutamate; m = myo‐inositol.; NAA = N‐acetyl aspartate. **Test:** ADAS‐cog = Alzheimer's Disease Assessment Scale; CDR = Clinical Dementia Rating; GDS = Geriatric Depression Scale; MoCA = Montreal Cognitive Assessment test; NMM= NeuroMET Memory Metric. **Voxel Locations:** ACG = Anterior Cingulate Gyrus; DACC = Dorsal Anterior Cingulate Cortex; H = hippocampus; L‐DLPFC = Left Dorsolateral Prefrontal Cortex; LH = Left Hippocampus; L‐LTL = Left Lateral Temporal Lobe; PCC = Posterior Cingulate Cortex; PCG = Posterior Cingulate Gyrus; PFC = Prefrontal Cortex; R‐DLPFC = Right Dorsolateral Prefrontal Cortex; RH = Right Hippocampus.

A limitation in data comparability stems from the fact that not all articles provide detailed information on clinical evaluations and the assessment scales employed. When such information was available, the neuropsychological and functional scales used (e.g., MMSE, CDR, DRS), the clinical diagnostic criteria applied (DSM, NINCDS‐ADRDA, Petersen, NIA‐AA), and the biomarkers assessed—including PET imaging, cerebrospinal fluid analyses, and plasma or genotyping markers—were reported.

## 3 Results

The use of MRS, DTI, and MRI has significantly enhanced our understanding of various aspects of AD pathophysiology. The studies included in this review have examined the role of non‐invasive MRI‐based techniques in the early diagnosis of AD—specifically in identifying individuals with amnestic mild cognitive impairment (aMCI) who are at higher risk of progressing to AD—thus offering new potential biomarkers in differential diagnosis and disease monitoring. The demand for accurate, specific, and early biomarkers of AD is steadily increasing in parallel with the rising number of patients and the aging global population.

In this narrative review we included 75 articles, meeting the inclusion criteria, dividing into: 24 MRS, 18 DTI, 33 MRI studies. We initially distinguish the results by focusing on each MRI‐based method; subsequently, we expose a combined picture.

MRS acquisition protocols: we included studies performed at 1.5 T (12 studies, 50%), 3 T (8 studies, 33%), 4 T (1 study, 4.16%) and 7 T (3 studies,12.5%) scanners.

Most MRS studies (74%) used the PRESS sequences; however, some metabolites (e.g., Glx, GABA, and GSH), due to short T2 relaxation times or spectral overlap, are difficult to measure at low magnetic fields. This is why additional sequences such as MEGA‐PRESS are used by some authors. Since they are difficult to measure; they are often considered as the sum of the metabolites (e.g., Glx = Gln + Glu).

At high magnetic fields, the chemical shift displacement error (CSDE) is more prominent [89]. For this reason, many authors used the semi‐LASER sequence.

We selected MRS studies with voxel sizes that vary depending on the considered brain region; they are most often located in the posterior cingulate cortex (PCC); voxels were also commonly located in the hippocampus, frontal lobe, and anterior cingulate cortex.

We selected studies that use water as a reference or that report ratios of individual metabolites to creatine, a stable metabolite commonly employed as an internal standard.

### 3.1 The Role of MRS in Early Diagnosis and Differential Assessment in AD

As early as 2004, Jones and Waldman [90] emphasized the value of MRS as an adjunct to structural imaging, in investigating the development of AD, tracking neurodegenerative progression, and evaluating treatment efficacy. Building on this, Targosz‐Gajniak [25] highlighted MRS as a promising predictor of cognitive decline and dementia progression, with significant potential to differentiate AD from CN individuals and to forecast the risk of progression from aMCI to AD.

MRS offers unique insights into the neurometabolic foundations of clinical heterogeneity and symptom severity within brain regions vulnerable to AD and other dementias. This non‐invasive technique allows in vivo quantification of brain metabolites present at millimolar concentrations, including N‐acetyl‐aspartate (NAA), myo‐inositol (mI), glutamate, glutamine, γ‐aminobutyric acid (GABA), total choline (composed mainly of phosphocholine and glycerophosphocholine), total creatine (composed of creatine and phosphocreatine, glutathione, lipids, and macromolecules).

The studies reviewed here commonly assess relative metabolite concentration, typically normalizing to total creatine, given its relative stability as a marker of energy metabolism. Deviations from physiological metabolite ratios may reflect a range of pathological processes implicated in AD, such as neuronal injury, glial activation, and neurotransmission alterations. These metabolic markers contribute to enhancing early diagnosis and monitoring of AD progression.

Overall, studied MRS agree in findings reduced NAA and increased mI levels in occipital and parietal regions proportional to AD severity. In disease progression, an increase in Cho was also observed. MRS highlights are summarized in Table 1.

#### 3.1.1 Single‐Voxel‐MRS: Posterior Cingulate Cortex (PCC) is the Most Frequently Affected Region in the Early Stages of AD

The included studies confirm that significant alterations in brain metabolites are observed in subjects with AD and MCI, compared to CN—particularly in key regions such as the posterior cingulate cortex (PCC), dorsolateral prefrontal cortex (DLPFC), hippocampus (H), and parietal areas [18, 21, 24, 28]. The PCC has received the most attention, as it is one of the earliest regions affected by AD and plays a crucial role in memory and visuospatial processing through its involvement in the Papez circuit [15, 16, 19, 21, 22].

#### 3.1.2 Alterations in NAA/Cr, mI/Cr as Early Biomarkers: Detectable Even in the Preclinical Phase

Already in 2000, Kantarci et al. [91] reported that MRS can detect elevated mI levels and reductions in NAA in subjects with aMCI, that is, preclinical phase of AD. These alterations are associated with disease progression, being present in subjects with MCI but becoming more pronounced in overt AD [29]. It has been demonstrated that, in AD and other dementias, metabolic changes involving NAA and mI detected by MRS can aid in diagnosis, identifying biomarkers not visible with other imaging modalities, and supporting their role as potential diagnostic markers. Changes in the levels of other metabolites such as Cho, Glu, Gln, and GABA are controversial because, to date, there are still technical limitations and a lack of standardization of MRS techniques [92]. Some authors claim that, in addition to a decrease in NAA and an increase in mI, there is also an increase in choline (Cho), suggesting that the latter may represent one of the first metabolic changes detectable by MRS [24]. In summary, MRS studies of the PCC in AD patients consistently report increased Cho/Cr ratio, increased mI/Cr ratio, and decreased NAA/Cr ratio. Various authors have proposed possible explanations for these alterations. Several authors [11], have shown that NAA, synthesized in neurons, is a reliable indicator of neuronal density and function; thus, its reduction reflects neuronal loss. Graff‐Radford and Kantarci [93] suggest that elevated Cho/Cr ratios may indicate glial activation and are predictive of cognitive decline in AD. Similarly, Tumati (2013) [22] reported that increased mI/Cr levels correlate with AD progression and the presence of neurofibrillary tangles and neuritic plaques in MCI patients [28, 29, 30, 33, 85]. A lateralization of the increase in myo‐inositol has been found in some cortical areas (the left side is more subject to variations than the right side) suggesting an asymmetric involvement in the disease [23]. Some studies have shown that the NAA/mI ratio represents a sensitive indicator in predicting the conversion from MCI to AD even up to 2 years before the onset of evident clinical symptoms [21, 30], and also able to predict the onset of the familial Alzheimer in presymptomatic mutation carriers [31], providing this ratio as valuable biomarkers for the early diagnosis to direct patients to proper therapy. Overall, these MRS‐detectable metabolic changes provide valuable biomarkers for the diagnosis and monitoring of AD‐related neurodegeneration.

#### 3.1.3 Metabolites's Change is Related to the Severity of the Symptoms and is Associated to the Progression of Disease

According to our review, there appears to be a link between neurometabolic alterations (NAA/Cr, mI/Cr, Cho/Cr) and disease progression, with specific behavioral symptoms (such as apathy, depression). MRS studies have consistently shown that these metabolic changes correlate with clinical symptoms and their severity in both AD and aMCI subjects. For example, Huang et al. [94] reported that reduced NAA/Cr levels in the occipital and parietal cortices are proportional to disease severity. Similarly, metabolic alterations are already detectable in MCI subjects but become more pronounced as the disease progresses to overt AD [95, 96]. Apathy is a common symptom associated with a higher risk of progression from aMCI to AD. Tumati and colleagues [22] investigated neurometabolic alterations underlying apathy in aMCI, focusing on brain regions involved in motivation and executive functions—including the posterior cingulate cortex (PCC), dorsal anterior cingulate cortex (DACC), right DLPFC, and right temporoparietal cortex (TPC). The authors also found that apathy severity was negatively correlated with Cho and myo‐inositol (mI) levels in the right TPC. Moreover, the NAA/mI ratio reduction (found in aMCI patients without apathy) was not observed in patients with apathy. On the contrary, glutamate + glutamine (Glx) levels were elevated in the DACC of aMCI patients with apathy compared to controls, but these levels decreased as depression scores increased. These findings suggest that aMCI with apathy is associated with distinct neurometabolic changes—particularly altered membrane integrity and glial dysfunctions in the right TPC—which differentiate it from aMCI without apathy. In line with this, Shinno et al. [97] reported that behavioral symptoms in dementia negatively correlate with NAA/Cr levels and positively correlate with mI/Cr ratios in the ACC. Overall, these studies indicate that MRS‐detectable neurometabolic alterations could be markers of disease severity and may also add valuable insights into the neurobiological mechanisms underlying specific behavioral symptoms in AD and aMCI.

### 3.2 Insights From DTI Studies: A Sensitive Tool for Early Diagnosis and Stratification Through Region‐Specific Detection of Gray and WM Degeneration

It is well established that parameters derived from DTI can detect microstructural abnormalities and provide valuable insights into WM integrity.

These measures are increasingly recognized as useful tools for differentiating between different types of dementia. In this chapter, we summarize, through DTI analysis, WM features that contribute to the progression of MCI to AD and describe their potential in identifying individuals at high risk for disease progression. Moreover, we also review the characteristics of WM alterations in AD and their relationship to cognitive functions.

DTI‐derived metrics, especially MD and FA, are promising biomarkers for evaluating the extent of WM damage in AD. FA reflects the degree of directional water diffusion; lower FA values indicate a loss of fiber coherence and integrity. Conversely, MD captures the overall magnitude of diffusion, with increased MD suggesting axonal degeneration or disruption of fiber structure. Additional informative parameters include apparent diffusion coefficient (ADC), relative anisotropy (RA), and eigenvalues (parallel and perpendicular to neuronal fiber).

The articles included in this review consistently demonstrate, from DTI‐based analysis, reduced FA and increased MD which correlate to WM alterations in AD patients as summarized in Table 2. These alterations are also useful for monitoring disease progression, facilitating early diagnosis during the preclinical phase, and aiding stratification.

DTI consistently reveals that both AD and MCI involve WM and GM alterations. MCI patients display intermediate DTI profiles between healthy controls and AD patients, reinforcing the idea that MCI represents a transitional state between normal aging and pathological decline. Notably, DTI can detect WM changes in asymptomatic or at‐risk individuals, underscoring its potential as an early biomarker for AD risk and progression.

Characteristic DTI findings in AD and MCI include increased MD and decreased FA, indicating neuronal and synaptic degeneration and WM disconnection. Such changes are particularly evident in the hippocampus, parahippocampal regions, posterior cingulum, splenium of the corpus callosum, fornix, precuneus, and prefrontal WM. Posterior WM tracts are especially vulnerable in early AD, with region‐specific damage patterns corresponding to cognitive deficits such as memory impairment, praxis difficulties, and executive dysfunction. For example, reduced FA in the MTA correlates with verbal memory deficits in MCI [98].

Numerous studies have examined regional differences in WM integrity across older adults with AD, MCI, and CN subjects. While early work (e.g., [45]) questioned the utility of DTI in detecting early MCI, subsequent studies [35, 40] identified significant differences in hippocampal MD in MCI patients, indicating that DTI can detect early microstructural changes even before clinical symptoms manifest.

AD pathology affects both cortical gray matter (GM) and WM. A recent study by Silva‐Rudberg et al. [34] showed an inverse relationship between synaptic loss—a primary correlation of cognitive decline—and microstructural GM changes in vulnerable regions, such as the hippocampus. Increased MD in GM correlated with lower synaptic density. Moreover, growing evidence suggests that WM microstructure is affected early in AD pathogenesis, although the precise mechanisms remain incompletely understood. DTI metrics also align with neuropathological staging (e.g., Braak stages for neurofibrillary tangles), further linking WM damage to tau pathology. Elevated CSF tau protein levels have been associated with WM microstructural changes, suggesting that axonal degeneration underlies early WM involvement in preclinical AD.

In summary, DTI studies demonstrate that WM alterations are integral to AD pathology, correlate closely with cognitive impairment, and offer valuable insights for early diagnosis and differential diagnosis. DTI‐derived parameters such as FA and MD hold significant promise as clinical and research biomarkers in dementia.

### 3.3 MRI Findings: Through MRI, Atrophy is Evident, Already in the Early Stages

According to Braak's theory, some brain regions are more affected by the neuropathological process underlying AD, namely neurofibrillary fibrillation and neuronal and synaptic loss. MRI‐detected atrophy is evident, already in the early stages, at the level of the entorhinal cortex (ERC) and hippocampus; in the intermediate stages, also frontal, parietal, and temporal cortex, thalamus, limbic cortex, and cingulate gyri. Later, in the late stages of the disease, atrophy also involves the cerebellum and occipital cortex (as confirmed by autopsy analyses).

Thus, structural neuroimaging studies have shown that different brain regions are progressively affected CN → aMCI → AD, supporting their role as early markers of neurodegeneration.

Volumetric results are shown in Table 3.

Cortical atrophy follows a progressive course, initially affecting the temporal lobes and subsequently involving the parietal and frontal regions [81]. Parietal atrophy has also been associated with visuospatial deficit [99]. Hippocampus's vulnerability increases with AD genetic factors, particularly the *APOE* region, which exert their influence primarily before or during the transition to MCI [51].

In progressive aMCI (aMCI‐P) compared to stable aMCI (aMCI‐S), greater atrophy is observed in the precuneus, posterior cingulate cortex, and frontal cortex, suggesting a distinct atrophic signature predictive of conversion to AD [80].

Importantly, ERC degeneration often precedes hippocampal volume loss and provides greater accuracy in differentiating MCI from AD than hippocampal volume alone [82]. Hippocampal volume progressively decreases, especially in individuals who later convert to AD [100] and is significantly correlated with lower cognitive scores on the MMSE, AVLT, BNT, and ADL tests [69]. Atrophy in the entorhinal and piriform cortex, regions associated with olfaction and episodic memory, has been observed as early as the aMCI stage [53].

Atrophy of the olfactory bulb and olfactory tract (OBT) are also present in the early stages of the disease [77]. Volume reduction in the piriform cortex and primary olfactory cortex has been linked to both aMCI and AD, with behavioral and cognitive correlates [71]. However, other studies suggest that OBT volume loss becomes significant only in advanced stages [55]. The basal forebrain cholinergic system (BFCS) also shows widespread volume loss in AD patients. In individuals with aMCI, selective atrophy is observed in the posterior basal nucleus of Meynert (NbM), while anteromedial regions remain relatively preserved [72]. Cerebellar volume is reduced by approximately 2.5% in advanced AD, although no significant differences are typically found between CN and MCI [63]. The brainstem, particularly the midbrain and locus coeruleus (LC), also shows atrophy, which has been linked to deficits in attention and executive functions [59, 70].

Atrophy in the amygdala, thalamus, putamen, and globus pallidus is commonly observed in both aMCI and AD patients [60] and is associated with cognitive decline and neuropsychiatric symptoms.

Tumati et al. [22] found distinct neural changes in the TPC of patients with apathy, conditions frequently observed in both aMCI and AD, from those in aMCI without apathy, which are related to reduced Cho and mI levels indicative of alterations in membranal and glial functions.

Combined metabolic and volumetric alterations (e.g., NAA/mI ratio and parahippocampal gyrus volume) can predict conversion to AD up to 2 years before clinical onset [21].

#### 3.3.1 MRS DTI And Structural MRI as Useful Tools to Study the Efficacy of Pharmacological and Not Pharmacological Treatments

Some studies highlight how MRS spectroscopy is useful not only to distinguish between AD, MCI, and controls but also to monitor the evolution of the disease and the response to treatments, with a possible clinical application in early diagnosis and follow‐up [25, 26]. In fact, MRS has been used as a method to verify the efficacy of pharmacological treatments, such as cholinesterase inhibitors (donepezil, rivastigmine) and galantamine or the efficacy of non‐pharmacological treatment such as transcranial magnetic stimulation or TMS [85]. These treatments have been associated with improvements in NAA levels, suggesting a possible neuroprotective effect or neuronal metabolic recovery [87] or neurogenesis [101].

Volumetric MRI and DTI have provided information that complements amyloid and tau PET imaging as well as functional MRI, offering additional markers of brain atrophy and microstructural injury [83, 84]. These findings further support their value for drug development and therapeutic clinical trials.

#### 3.3.2 Current Technical Limitations and the Need for Standardized Protocols

To date, alterations in the levels of certain metabolites—such as (Cho, discussed above), glutamate (Glu, the main excitatory neurotransmitter in the central nervous system), glutamine (Gln, a precursor of glutamate), and gamma‐aminobutyric acid (GABA, a key inhibitory neurotransmitter)—remain somewhat controversial due to technical limitations and the lack of standardized MRS acquisition protocols [92].

In this review, we included MRS studies that employed acquisition sequences such as STimulated Echo Acquisition Mode (STEAM), Point Resolved Spectroscopy (PRESS), and semi‐LASER (s‐LASER), with echo times ranging from 25 to 272 ms. Repetition time and echo time were considered for the spectroscopy studies, as they are important for the interpretation of the resulting metabolic information. In contrast, these acquisition parameters were not considered in the discussion of findings from MRI and DTI studies. Often, these are multicenter studies, where data acquisitions were performed with varying scanners, protocols, and parameters. We included single‐voxel localization methods only to describe the quantitative results, which are more robust and comparable between different centers.

These methodological variations contribute to inconsistent findings across studies. However, the use of high‐field MRS (≥ 3 Tesla) and the adoption of standardized, advanced acquisition protocols are expected to overcome many of these limitations, enabling more accurate and reproducible quantification of a broader range of metabolites. In fact, the lack of standardized protocols for the acquisition and analysis of MRS, already highlighted in the early 80s [102], is still present and underlines the importance of the collaborative work of international societies in defining standardized acquisition protocols for quantitative MRS. Considering recent Consensus Papers [89, 103] which give the minimum requirements to be adopted in quantitative MRS studies, we are confident that in the near future MRS may be useful to define new and specific diagnostic and prognostic biomarkers. This, in turn, could significantly enhance the clinical utility of MRS in the early diagnosis and monitoring of Alzheimer's disease and other neurodegenerative disorders.

Whenever possible, it is recommended to apply quantitative protocols which include the use of:

1. Semi‐Laser sequence instead of PRESS (and semi‐Special instead of STEAM) at magnetic field larger than 3 T,
2. High quality automated shimming,
3. Short echo time (to minimize the signal loss due to metabolite T2s),
4. Long repetition time (to minimize the effect of metabolite T1s),
5. T2‐corrected water signal as internal reference and
6. Fitting routines which combine metabolites, lipids and macromolecules basis‐set for spectral analysis.

The evaluation of differences between different types of dementia using multimodal MRI represents the future goal of scientific research, aimed at identifying early diagnostic biomarkers, monitoring disease progression, and response to innovative therapies.

Issues such as protocol heterogeneity, the lack of standardized acquisition and processing pipelines, the influence of free‐water effects on DTI metrics—particularly pronounced in atrophic regions—and substantial inter‐site variability have limited the clinical translatability of diffusion‐based biomarkers. A deeper examination of how these methodological inconsistencies distort quantitative metrics and contribute to variability in effect sizes is warranted before the potential of advanced diffusion techniques and volumetric measures can be fully exploited to generate reliable and clinically actionable biomarkers for neurodegenerative diseases.

### 3.4 Integration of Multimodal MRI Biomarkers Among Themselves and With Established Biomarker Modalities

Integrating MRS, DTI, and structural MRI allows clinicians and researchers to combine metabolic measures (e.g., NAA and mI changes detectable with MRS), microstructural markers (e.g., reduced FA or increased free‐water fraction in white‐matter tracts on DTI), and macrostructural patterns of atrophy to improve early detection and biological characterization of neurodegenerative diseases. DTI detects microstructural degeneration in Alzheimer's disease even when structural MRI appears normal, and MRS abnormalities (e.g., ↓NAA/Cre, ↑mI) can reveal metabolic dysfunction in regions without visible atrophy, while volumetry differentiates phenotypes such as hippocampal‐predominant versus other subtype of AD. This multimodal approach enhances diagnostic accuracy, refines prognostic stratification, and increases sensitivity for monitoring progression and treatment effects, thereby improving clinical translation [104].

The multimodal MRI approach enables a more precise linkage with PET, CSF, and blood‐based biomarkers: MRS connects amyloid and tau deposition to in vivo metabolic dysfunction, DTI uncovers microstructural white‐matter alterations invisible to fluid biomarkers or amyloid PET and supports differential diagnosis, while structural MRI, when combined with amyloid or tau PET, contextualizes molecular pathology by delineating the extent and pattern of tissue loss across dementia syndromes. Application of advanced analytic approaches—such as machine learning frameworks and integrative modeling—will enable the combination of neuroimaging measures with genetic profiles and fluid biomarkers to understand, classify, and stage the various forms of dementia [105].

### 3.5 Assessment of Clinical Heterogeneity Among CN and Participants With MCI and AD

In this review, we included 75 articles. These studies revealed substantial clinical heterogeneity among subjects, which we will address in this section to provide a realistic, critical, and more meaningful estimate of the potential clinical applicability of MR techniques for the early diagnosis of AD. Our analysis focused on AD and amnestic progressive MCI, as these groups are more directly related to AD‐specific neurobiological mechanisms and are most relevant for identifying early biomarkers predictive of AD conversion. Stable and non‐AD MCI populations, although clinically relevant, were beyond the specific scope of our research question.

Clinically, it is well established that there is considerable interindividual variability in neuroscience, evident both in cognitive performance and in metabolic and structural patterns.

This variability makes it challenging to generalize diagnostic and prognostic findings to large populations and to implement universal clinical guidelines for diagnosis and prognosis.

We address clinical variability, because it may influence the observed results from cognitive (obtained from neuropsychological tests), metabolic (with MRS), microstructural (with DTI), or structural (with MRI) perspectives. Indeed, the studies included in this review indicate that alterations are caused by AD progression in different brain regions, but with inconsistent results, as neurodegeneration does not affect subjects homogeneously nor the same brain areas. Furthermore, studies may present differential subtypes in terms of disease stage (mild vs. moderate vs. severe AD), MCI subtypes (e.g., amnestic vs. non‐amnestic MCI), presence of symptoms (including apathy, depression), or the use of pharmacology (galantamine, donepezil, rivastigmine) or non‐pharmacological (including exercise, TMS) treatments. Some studies (e.g., [20, 21]) show progressive metabolic patterns at different clinical stages of the disease or depending on the specific symptom subtype (e.g., [22] distinguishes aMCI with/without apathy). This would also lead to a heterogeneous response to therapeutic treatments: for example, [17, 85, 86, 87] demonstrate that therapeutic treatments affect sub‐hippocampal volume differently depending on the subject's cognitive risk.

Evaluating factors (including subjects' age, cultural level and years of education, clinical severity, and performance on neuropsychological tests) is essential to contextualize the results obtained, which is why each article included in this review includes a demographic data collection in its materials and methods. Articles that present a clear collection of this data are deemed most suitable for inclusion in this review.

Specifically, the studies included here present a comprehensive collection of demographic data, including age, sex, clinical assessments of neuropsychiatric symptoms and daily activity (measured using the Mini‐Mental State Examination—MMSE—, The Rey Auditory Verbal Learning Test, and Alzheimer's Disease Assessment Scale).

Regarding age, subjects were included in a general age range, often between 65 and 80 years; however, studies on younger populations (e.g., familial genetic mutations—PSEN mutation) were also included. The studies, which also reported the percentage distribution of sex, tended to be gender balanced. Additionally, MMSE and CDR (Clinical Dementia Rating) scores were collected for each study. Typically, studies characterize: CN with an MMSE ≥ 28 and CDR = 0; MCI with an MMSE between 24 and 30 and a CDR = 0.5; and AD with an MMSE < 20 and a CDR = 1 (mild), CDR = 2 (moderate), and CDR = 3 (severe).

Due to the high clinical heterogeneity, we believe it is essential for future studies to define more stringent inclusion criteria and standardize more accurate methodologies, which could lead to a more accurate and universally accepted use of MR‐techniques in the clinical monitoring of the disease.

Finally, because a single measurement may not be sufficient to capture the complexity of the subject's cognitive, metabolic, and structural profile, we suggest using a combination of MR techniques in conjunction with neuropsychological tests, as the integration of multiple tools allows for better understanding of interpersonal variability. This procedure allows for a more sensitive and specific assessment of the alterations present at different stages of the disease and can improve diagnostic accuracy and disease predictability.

## 4 Conclusions

**FIGURE 2:**
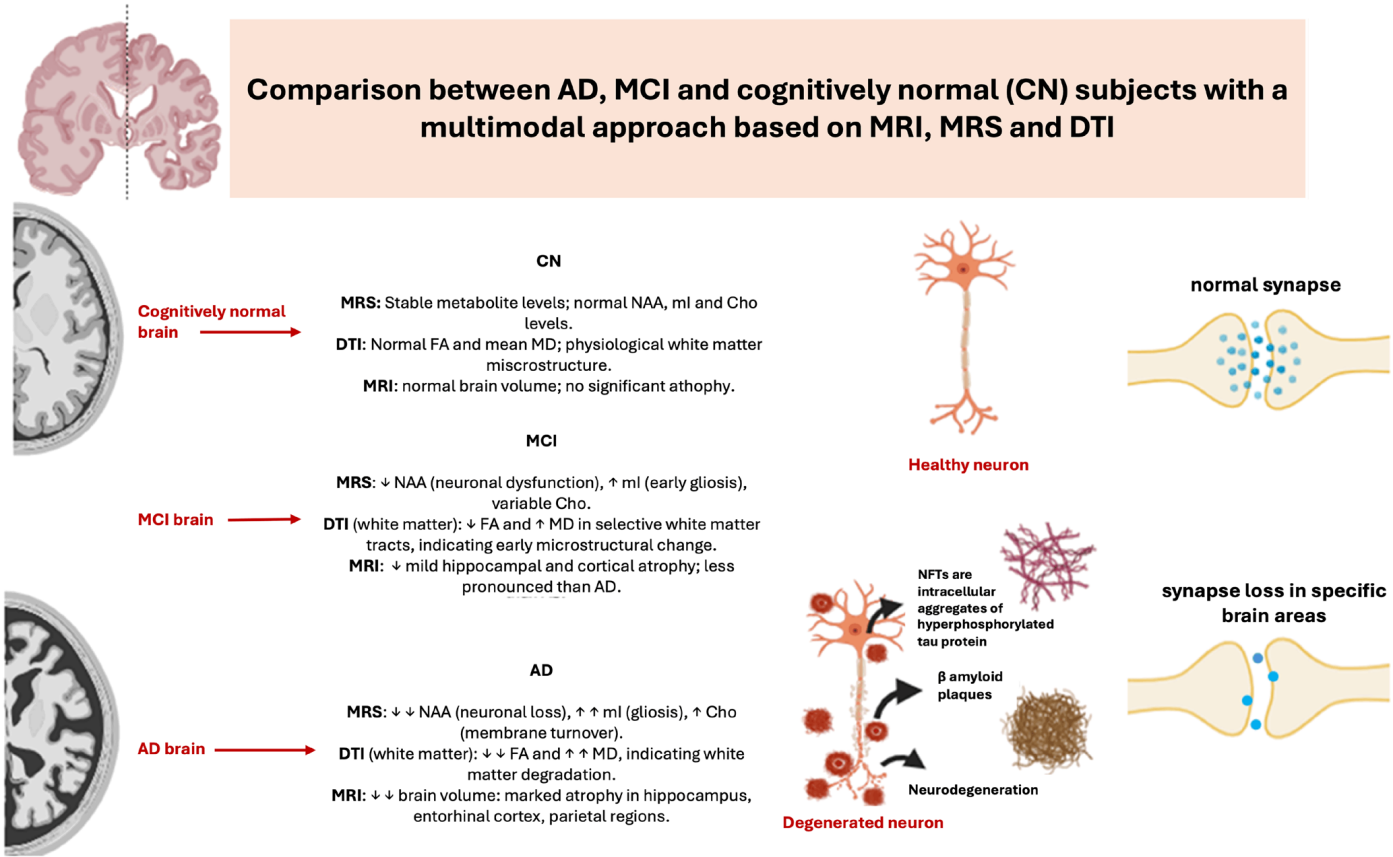
Comparison between AD, MCI and CN subjects. Comparison between AD, MCI and CN subjects. Comparison of healthy, MCI, and AD brains using a multimodal approach based on MRI, MRS, and DTI. Healthy subjects have normal brain volumes, intact white matter microstructure, and stable metabolic levels. MCI shows reduced NAA, initial signs of gliosis (increased mI), microstructural alterations in white matter (increased MD and reduced FA), and mild hippocampal and cortical atrophy. In AD subjects, the alterations become more pronounced: Significant neuronal loss with a marked reduction in NAA, increased myo‐inositol and choline, white matter degradation, and severe atrophy of the hippocampus and cortical areas, accompanied by amyloid plaques, neurofibrillary tangles, and synaptic loss.

The findings of this narrative review, summarized in Figure 2, indicate that a multimodal approach based on multimodal MRI may serve as a valuable tool for enhancing early diagnosis and patient stratification, alongside biological and clinical assessments. Furthermore, it could support monitoring the progression of AD. Metabolic alterations detected by MRS—decrease in NAA, increases in mI concentrations—could be measured already in the preclinical phases and correlate with both disease progression and specific behavioral symptoms. DTI techniques show early alterations in the WM microstructure (increases in MD and decrease in FA), consistent with the axonal and synaptic damage observed in AD pathophysiology, while regional atrophy measurable by anatomic MRI confirms the gradual involvement of cortical and subcortical areas (the first areas affected are: ERC and hippocampus).

In conclusion, the most important application of volumetric MRI, DTI, and MRS is their ability to provide complementary information to amyloid and tau PET imaging and to functional MRI, serving as markers of microstructural damage and metabolic dysfunction. This integrated approach is particularly valuable for drug development and therapeutic trials.

Methodological limitations, especially the lack of standardized protocols, limit the potential value of these techniques in providing sensitive and specific biomarkers for diagnosis, stratification, and monitoring of AD. Future developments should aim at greater integration between imaging parameters and the definition of shared guidelines, with great importance to standardized protocols, to further improve diagnostic accuracy and the personalization of therapeutic interventions.

## Conflicts of Interest

The authors declare no conflicts of interest.

## References

[1] 2025 Alzheimer's Disease Facts and Figures, Alzheimer's & Dementia 21 (2025): 21.

[2] C. R. Jack , S. J. Andrews , T. G. Beach , et al., “Revised Criteria for the Diagnosis and Staging of Alzheimer's Disease,” Nature Medicine 30 (2024): 2121–2124. 10.1038/s41591-024-02988-7 PMC11630478 38942991

[3] G. M. Mckhann , D. S. Knopman , H. Chertkow , et al., “The Diagnosis of Dementia due to Alzheimer's Disease: Recommendations From the National Institute on Aging‐Alzheimer's Association Workgroups on Diagnostic Guidelines for Alzheimer's Disease NIH Public Access,” Alzheimer's & Dementia 7 (2011): 263–269. 10.1016/j.jalz.2011.03.005 PMC3312024 21514250

[4] M. S. Albert , S. T. DeKosky , D. Dickson , et al., “The Diagnosis of Mild Cognitive Impairment due to Alzheimer's Disease: Recommendations From the National Institute on Aging‐Alzheimer's Association Workgroups on Diagnostic Guidelines for Alzheimer's Disease,” Alzheimer's & Dementia 7 (2011): 270–279. 10.1016/j.jalz.2011.03.008 PMC3312027 21514249

[5] R. C. Petersen , R. Doody , A. Kurz , et al., “Current Concepts in Mild Cognitive Impairment,” Archives of Neurology 58, no. 12 (2001): 1985–1992. 11735772 10.1001/archneur.58.12.1985

[6] R. C. Petersen , G. E. Smith , S. C. Waring , R. J. Ivnik , E. G. Tangalos , and E. Kokmen , “Mild Cognitive Impairment Clinical Characterization and Outcome,” Archives of Neurology 56, no. 3 (1999): 303–308. 10190820 10.1001/archneur.56.3.303

[7] G. McKhann , D. Drachman , M. Folstein , R. Katzman , D. Price , and E. M. Stadlan , “Clinical Diagnosis of Alzheimer's Disease: Report of the NINCDS‐ADRDA Work Group⋆ Under the Auspices of Department of Health and Human Services Task Force on Alzheimer's Disease,” Neurology 34 (1984): 939–944. 6610841 10.1212/wnl.34.7.939

[8] P. Tideman , L. Karlsson , O. Strandberg , et al., “Primary Care Detection of Alzheimer's Disease Using a Self‐Administered Digital Cognitive Test and Blood Biomarkers,” Nature Medicine 31 (2025): 4131–4139. 10.1038/s41591-025-03965-4 PMC12705462 40954312

[9] E. Fabrizi , A. Ancidoni , N. Locuratolo , et al., “The Italian Guideline on Diagnosis and Treatment of Dementia and Mild Cognitive Impairment,” Age and Ageing 53 (2024): afae250. 39544104 10.1093/ageing/afae250 PMC11564805

[10] E. Spina , R. R. Ferrari , E. Pellegrini , et al., “Mitochondrial Alterations, Oxidative Stress, and Therapeutic Implications in Alzheimer's Disease: A Narrative Review,” Cells 14 (2025): 229. 39937020 10.3390/cells14030229 PMC11817193

[11] P. Tofts , Quantitative MRI of the Brain: Measuring Changes Caused by Disease (Wiley, 2003).

[12] D. R. Chhetri , “Myo‐Inositol and Its Derivatives: Their Emerging Role in the Treatment of Human Diseases,” Frontiers in Pharmacology 10 (2019): 1172. 31680956 10.3389/fphar.2019.01172 PMC6798087

[13] M. Lv , K. Zhan , J. Yang , et al., “Analysis of Advanced Diffusion Models Assessing White Matter Microstructure in Alzheimer's Disease,” Scientific Reports 15 (2025): 15. 40683919 10.1038/s41598-025-09412-1 PMC12276235

[14] B. Dubois , C. A. F. von Arnim , N. Burnie , S. Bozeat , and J. Cummings , “Biomarkers in Alzheimer's Disease: Role in Early and Differential Diagnosis and Recognition of Atypical Variants,” Alzheimer's Research & Therapy 15 (2023): 175. 10.1186/s13195-023-01314-6 PMC10571241 37833762

[15] L. Göschel , A. Dell'Orco , A. Fillmer , et al., “Plasma p‐tau181 and GFAP Reflect 7T MR‐Derived Changes in Alzheimer's Disease: A Longitudinal Study of Structural and Functional MRI and MRS,” Alzheimer's & Dementia 20 (2024): 8684–8699. 10.1002/alz.14318 PMC11667506 39558898

[16] C. W. Davies‐Jenkins , C. I. Workman , K. E. Hupfeld , et al., “Multimodal Investigation of Neuropathology and Neurometabolites in Mild Cognitive Impairment and Late‐Life Depression With 11C‐PiB Beta‐Amyloid PET and 7T Magnetic Resonance Spectroscopy,” Neurobiology of Aging 142 (2024): 27–40. 39111221 10.1016/j.neurobiolaging.2024.06.003 PMC11916921

[17] W. A. J. Vints , J. Šeikinaitė , E. Gökçe , et al., “Resistance Exercise Effects on Hippocampus Subfield Volumes and Biomarkers of Neuroplasticity and Neuroinflammation in Older Adults With Low and High Risk of Mild Cognitive Impairment: A Randomized Controlled Trial,” Geroscience 46 (2024): 3971–3991. 38478179 10.1007/s11357-024-01110-6 PMC11226571

[18] K. Valatkevičienė , O. Levin , M. Šarkinaitė , et al., “N‐Acetyl‐Aspartate and Myo‐Inositol as Markers of White Matter Microstructural Organization in Mild Cognitive Impairment: Evidence From a DTI‐1H‐MRS Pilot Study,” Diagnostics 13 (2023): 13. 10.3390/diagnostics13040654 PMC9955118 36832141

[19] F. Kara , J. M. Joers , D. K. Deelchand , et al., “1H MR Spectroscopy Biomarkers of Neuronal and Synaptic Function Are Associated With Tau Deposition in Cognitively Unimpaired Older Adults,” Neurobiology of Aging 112 (2022): 16–26. 35038671 10.1016/j.neurobiolaging.2021.12.010 PMC8976711

[20] D. Wong , S. Atiya , J. Fogarty , et al., “Reduced Hippocampal Glutamate and Posterior Cingulate N‐Acetyl Aspartate in Mild Cognitive Impairment and Alzheimer's Disease Is Associated With Episodic Memory Performance and White Matter Integrity in the Cingulum: A Pilot Study,” Journal of Alzheimer's Disease 73 (2020): 1385–1405. 10.3233/JAD-190773 31958093

[21] M. Mitolo , M. Stanzani‐Maserati , S. Capellari , et al., “Predicting Conversion From Mild Cognitive Impairment to Alzheimer's Disease Using Brain 1 H‐MRS and Volumetric Changes: A Two‐ Year Retrospective Follow‐Up Study,” NeuroImage: Clinical 23 (2019): 101843. 31071594 10.1016/j.nicl.2019.101843 PMC6506639

[22] S. Tumati , E. M. Opmeer , J. B. C. Marsman , et al., “Lower Choline and Myo‐Inositol in Temporo‐Parietal Cortex Is Associated With Apathy in Amnestic MCI,” Frontiers in Aging Neuroscience 10, no. APR (2018): 106. 29706886 10.3389/fnagi.2018.00106 PMC5909116

[23] Z. Guo , X. Liu , H. Hou , et al., “1H‐MRS Asymmetry Changes in the Anterior and Posterior Cingulate Gyrus in Patients With Mild Cognitive Impairment and Mild Alzheimer's Disease,” Comprehensive Psychiatry 69 (2016): 179–185. 27423359 10.1016/j.comppsych.2016.06.001

[24] J. X. Zou , M. J. Wang , X. J. Lei , and X. G. Chen , “3.0T MRI Arterial Spin Labeling and Magnetic Resonance Spectroscopy Technology in the Application of Alzheimer's Disease,” Experimental Gerontology 60 (2014): 31–36. 25220149 10.1016/j.exger.2014.09.009

[25] M. G. Targosz‐Gajniak , J. S. Siuda , M. M. Wicher , et al., “Magnetic Resonance Spectroscopy as a Predictor of Conversion of Mild Cognitive Impairment to Dementia,” Journal of the Neurological Sciences 335 (2013): 58–63. 24035276 10.1016/j.jns.2013.08.023

[26] T. Wang , S. Xiao , X. Li , et al., “Using Proton Magnetic Resonance Spectroscopy to Identify Mild Cognitive Impairment,” International Psychogeriatrics 24 (2012): 19–27. 21676281 10.1017/S1041610211000962

[27] T. Watanabe , A. Shiino , and I. Akiguchi , “Absolute Quantification in Proton Magnetic Resonance Spectroscopy Is Useful to Differentiate Amnesic Mild Cognitive Impairment From Alzheimer's Disease and Healthy Aging,” Dementia and Geriatric Cognitive Disorders 30 (2010): 71–77. 20689286 10.1159/000318750

[28] K. Kantarci , D. S. Knopman , D. W. Dickson , et al., “Alzheimer Disease: Postmortem Neuropathologic Correlates of Antemortem 1H MR Spectroscopy Metabolite Measurements,” Radiology 248 (2008): 210–220. 18566174 10.1148/radiol.2481071590 PMC2735577

[29] K. Kantarci , S. D. Weigand , R. C. Petersen , et al., “Longitudinal 1H MRS Changes in Mild Cognitive Impairment and Alzheimer's Disease,” Neurobiology of Aging 28 (2007): 1330–1339. 16860440 10.1016/j.neurobiolaging.2006.06.018 PMC2766807

[30] A. Metastasio , P. Rinaldi , R. Tarducci , et al., “Conversion of MCI to Dementia: Role of Proton Magnetic Resonance Spectroscopy,” Neurobiology of Aging 27 (2006): 926–932. 15936850 10.1016/j.neurobiolaging.2005.05.002

[31] A. K. Godbolt , A. D. Waldman , D. G. Macmanus , et al., “MRS Shows Abnormalities Before Symptoms in Familial Alzheimer Disease,” Neurology 66 (2006): 718–722. 16534109 10.1212/01.wnl.0000201237.05869.df

[32] N. Ackl , M. Ising , Y. A. Schreiber , M. Atiya , A. Sonntag , and D. P. Auer , “Hippocampal Metabolic Abnormalities in Mild Cognitive Impairment and Alzheimer's Disease,” Neuroscience Letters 384 (2005): 23–28. 15905028 10.1016/j.neulet.2005.04.035

[33] K. Kantarci , “Proton MRS in Mild Cognitive Impairment,” Journal of Magnetic Resonance Imaging 37 (2013): 770–777. 23526756 10.1002/jmri.23800 PMC3609038

[34] J. A. Silva‐Rudberg , E. Salardini , R. S. O'Dell , et al., “Assessment of Gray Matter Microstructure and Synaptic Density in Alzheimer's Disease: A Multimodal Imaging Study With DTI and SV2A PET,” American Journal of Geriatric Psychiatry 32 (2024): 17–28. 10.1016/j.jagp.2023.08.002 PMC10840732 37673749

[35] K. Brueggen , M. Dyrba , A. Cardenas‐Blanco , et al., “Structural Integrity in Subjective Cognitive Decline, Mild Cognitive Impairment and Alzheimer's Disease Based on Multicenter Diffusion Tensor Imaging,” Journal of Neurology 266 (2019): 2465–2474. 31227891 10.1007/s00415-019-09429-3

[36] K. Kantarci , M. E. Murray , C. G. Schwarz , et al., “White‐Matter Integrity on DTI and the Pathologic Staging of Alzheimer's Disease,” Neurobiology of Aging 56 (2017): 172–179. 28552181 10.1016/j.neurobiolaging.2017.04.024 PMC5523458

[37] C. D. Mayo , E. L. Mazerolle , L. Ritchie , J. D. Fisk , and J. R. Gawryluk , “Longitudinal Changes in Microstructural White Matter Metrics in Alzheimer's Disease,” NeuroImage: Clinical 13 (2017): 330–338. 28066707 10.1016/j.nicl.2016.12.012 PMC5200876

[38] M. M. Mielke , O. C. Okonkwo , K. Oishi , et al., “Fornix Integrity and Hippocampal Volume Predict Memory Decline and Progression to AD,” Alzheimer's & Dementia 8 (2012): 105–113. 10.1016/j.jalz.2011.05.2416 PMC3305232 22404852

[39] S. J. Teipel , M. Wegrzyn , T. Meindl , et al., “Anatomical MRI and DTI in the Diagnosis of Alzheimer's Disease: A European Multicenter Study,” Journal of Alzheimer's Disease 31 (2012): S33–S47. 10.3233/JAD-2012-112118 22992380

[40] Y. Liu , G. Spulber , K. K. Lehtimäki , et al., “Diffusion Tensor Imaging and Tract‐Based Spatial Statistics in Alzheimer's Disease and Mild Cognitive Impairment,” Neurobiology of Aging 32 (2011): 1558–1571. 19913331 10.1016/j.neurobiolaging.2009.10.006

[41] K. Kantarci , R. Avula , M. L. Senjem , et al., “Dementia With Lewy Bodies and Alzheimer Disease Neurodegenerative Patterns Characterized by DTI,” Neurology 74 (2010): 1814–1821. 20513818 10.1212/WNL.0b013e3181e0f7cf PMC2882217

[42] M. Sjöbeck , C. Elfgren , E. M. Larsson , et al., “Alzheimer's Disease (AD) and Executive Dysfunction. A Case‐Control Study on the Significance of Frontal White Matter Changes Detected by Diffusion Tensor Imaging (DTI),” Archives of Gerontology and Geriatrics 50 (2010): 260–266. 19419776 10.1016/j.archger.2009.03.014

[43] H. Cho , W. Y. Dong , M. S. Young , et al., “Abnormal Integrity of Corticocortical Tracts in Mild Cognitive Impairment: A Diffusion Tensor Imaging Study,” Journal of Korean Medical Science 23 (2008): 477–483. 18583886 10.3346/jkms.2008.23.3.477 PMC2526517

[44] V. Kavcic , H. Ni , T. Zhu , J. Zhong , and C. J. Duffy , “White Matter Integrity Linked to Functional Impairments in Aging and Early Alzheimer's Disease,” Alzheimer's & Dementia 4 (2008): 381–389. 10.1016/j.jalz.2008.07.001 PMC2653423 19012862

[45] R. Stahl , O. Dietrich , S. J. Teipel , H. Hampel , M. F. Reiser , and S. O. Schoenberg , “White Matter Damage in Alzheimer Disease and Mild Cognitive Impairment: Assessment With Diffusion‐Tensor MR Imaging and Parallel Imaging Techniques,” Radiology 243 (2007): 483–492. 17456872 10.1148/radiol.2432051714

[46] M. J. Firbank , A. M. Blamire , M. S. Krishnan , et al., “Diffusion Tensor Imaging in Dementia With Lewy Bodies and Alzheimer's Disease,” Psychiatry Research: Neuroimaging 155 (2007): 135–145. 10.1016/j.pscychresns.2007.01.001 17408930

[47] Y. Zhang , N. Schuff , and G. Jahng , “Diffusion Tensor Imaging of Cingulum Fibers in Mild Cognitive Impairment and Alzheimer Disease,” Neurology 68, no. 1 (2007): 13–19. 17200485 10.1212/01.wnl.0000250326.77323.01 PMC1941719

[48] D. Sydykova , R. Stahl , O. Dietrich , et al., “Fiber Connections Between the Cerebral Cortex and the Corpus Callosum in Alzheimer's Disease: A Diffusion Tensor Imaging and Voxel‐Based Morphometry Study,” Cerebral Cortex 17 (2007): 2276–2282. 17164468 10.1093/cercor/bhl136

[49] O. Naggara , C. Oppenheim , D. Rieu , et al., “Diffusion Tensor Imaging in Early Alzheimer's Disease,” Psychiatry Research: Neuroimaging 146 (2006): 243–249. 10.1016/j.pscychresns.2006.01.005 16520023

[50] S. Xie , J. X. Xiao , G. L. Gong , et al., “Voxel‐Based Detection of White Matter Abnormalities in Mild Alzheimer Disease,” Neurology 66 (2006): 1845–1849. 16801648 10.1212/01.wnl.0000219625.77625.aa

[51] N. Vilor‐Tejedor , A. Rodrigo , P. Genius , et al., “Genetic Drivers of Hippocampal Atrophy Highlight the Role of APOE Functional Variants and AD Polygenicity in Mild Cognitive Impairment,” NeuroImage: Clinical 48 (2025): 103889. 41092763 10.1016/j.nicl.2025.103889 PMC12552153

[52] S. Langella , F. Lopera , A. Baena , et al., “Depressive Symptoms and Hippocampal Volume in Autosomal Dominant Alzheimer's Disease,” Alzheimer's & Dementia 20 (2024): 986–994. 10.1002/alz.13501 PMC10916972 37837524

[53] D. Steinbart , S. N. Yaakub , M. Steinbrenner , et al., “Automatic and Manual Segmentation of the Piriform Cortex: Method Development and Validation in Patients With Temporal Lobe Epilepsy and Alzheimer's Disease,” Human Brain Mapping 44 (2023): 3196–3209. 37052063 10.1002/hbm.26274 PMC10171523

[54] S. Mathew , D. Wudunn , D. D. Mackay , et al., “Association of Brain Volume and Retinal Thickness in the Early Stages of Alzheimer's Disease,” Journal of Alzheimer's Disease 91 (2023): 743–752. 10.3233/JAD-210533 PMC9990456 36502316

[55] S. E. Carnemolla , F. Kumfor , C. T. Liang , D. Foxe , R. M. Ahmed , and O. Piguet , “Olfactory Bulb Integrity in Frontotemporal Dementia and Alzheimer's Disease,” Journal of Alzheimer's Disease 89 (2022): 51–66. 10.3233/JAD-220080 35848020

[56] A. S. Bernstein , S. Z. Rapcsak , M. Hornberger , and M. Saranathan , “Structural Changes in Thalamic Nuclei Across Prodromal and Clinical Alzheimer's Disease,” Journal of Alzheimer's Disease 82 (2021): 361–371. 10.3233/JAD-201583 34024824

[57] L. A. Van De Mortel , R. M. Thomas , and G. A. Van Wingen , “Grey Matter Loss at Different Stages of Cognitive Decline: A Role for the Thalamus in Developing Alzheimer's Disease,” Journal of Alzheimer's Disease 83 (2021): 705–720. 10.3233/JAD-210173 PMC8543264 34366336

[58] O. Iritani , T. Okuno , T. Miwa , et al., “Olfactory‐Cognitive Index Distinguishes Involvement of Frontal Lobe Shrinkage, as in Sarcopenia From Shrinkage of Medial Temporal Areas, and Global Brain, as in Kihon Checklist Frailty/Dependence, in Older Adults With Progression of Normal Cognition to Alzheimer's Disease,” Geriatrics & Gerontology International 21 (2021): 291–298. 33465821 10.1111/ggi.14128 PMC7986338

[59] S. Dutt , Y. Li , M. Mather , and D. A. Nation , “Brainstem Volumetric Integrity in Preclinical and Prodromal Alzheimer's Disease,” Journal of Alzheimer's Disease 77 (2020): 1579–1594. 10.3233/JAD-200187 PMC7868064 32925030

[60] H. S. Yoo , E. C. Lee , S. J. Chung , et al., “Effects of Alzheimer's Disease and Lewy Body Disease on Subcortical Atrophy,” European Journal of Neurology 27 (2020): 318–326. 31487756 10.1111/ene.14080

[61] N. Boublay , R. Bouet , J. M. Dorey , et al., “Brain Volume Predicts Behavioral and Psychological Symptoms in Alzheimer's Disease,” Journal of Alzheimer's Disease 73 (2020): 1343–1353. 10.3233/JAD-190612 31903989

[62] E. Vuoksimaa , L. K. McEvoy , D. Holland , C. E. Franz , and W. S. Kremen , “Modifying the Minimum Criteria for Diagnosing Amnestic MCI to Improve Prediction of Brain Atrophy and Progression to Alzheimer's Disease,” Brain Imaging and Behavior 14 (2020): 787–796. 30511118 10.1007/s11682-018-0019-6 PMC7275013

[63] H. Tabatabaei‐Jafari , E. Walsh , M. E. Shaw , and N. Cherbuin , “The Cerebellum Shrinks Faster Than Normal Ageing in Alzheimer's Disease but Not in Mild Cognitive Impairment,” Human Brain Mapping 38 (2017): 3141–3150. 28321950 10.1002/hbm.23580 PMC5426955

[64] L. Agüera‐Ortiz , J. A. Hernandez‐Tamames , P. Martinez‐Martin , et al., “Structural Correlates of Apathy in Alzheimer's Disease: A Multimodal MRI Study,” International Journal of Geriatric Psychiatry 32 (2017): 922–930. 27428560 10.1002/gps.4548

[65] E. Makovac , L. Serra , B. Spanò , et al., “Different Patterns of Correlation Between Grey and White Matter Integrity Account for Behavioral and Psychological Symptoms in Alzheimer's Disease,” Journal of Alzheimer's Disease 50 (2016): 591–604. 10.3233/JAD-150612 26836635

[66] M. Torso , L. Serra , G. Giulietti , et al., “Strategic Lesions in the Anterior Thalamic Radiation and Apathy in Early Alzheimer's Disease,” PLoS One 10 (2015): e0124998. 25932637 10.1371/journal.pone.0124998 PMC4416903

[67] K. Brueggen , M. Dyrba , F. Barkhof , et al., “Basal Forebrain and Hippocampus as Predictors of Conversion to Alzheimer's Disease in Patients With Mild Cognitive Impairment‐a Multicenter DTI and Volumetry Study,” Journal of Alzheimer's Disease 48 (2015): 197–204. 10.3233/JAD-150063 26401940

[68] E. L. Elcombe , J. Lagopoulos , S. L. Duffy , et al., “Hippocampal Volume in Older Adults at Risk of Cognitive Decline: The Role of Sleep, Vascular Risk, and Depression,” Journal of Alzheimer's Disease 44 (2015): 1279–1290. 10.3233/JAD-142016 25408219

[69] G. P. Peng , Z. Feng , F. P. He , et al., “Correlation of Hippocampal Volume and Cognitive Performances in Patients With Either Mild Cognitive Impairment or Alzheimer's Disease,” CNS Neuroscience & Therapeutics 21 (2015): 15–22. 25146658 10.1111/cns.12317 PMC6495306

[70] J. H. Lee , J. Ryan , C. Andreescu , H. Aizenstein , and H. K. Lim , “Brainstem Morphological Changes in Alzheimer's Disease,” Neuroreport 26 (2015): 411–415. 25830491 10.1097/WNR.0000000000000362 PMC4415522

[71] M. M. Vasavada , J. Wang , P. J. Eslinger , et al., “Olfactory Cortex Degeneration in Alzheimer's Disease and Mild Cognitive Impairment,” Journal of Alzheimer's Disease 45 (2015): 947–958. 10.3233/JAD-141947 25633674

[72] I. Kilimann , M. Grothe , H. Heinsen , et al., “Subregional Basal Forebrain Atrophy in Alzheimer's Disease: A Multicenter Study,” Journal of Alzheimer's Disease 40 (2014): 687–700. 10.3233/JAD-132345 PMC4120953 24503619

[73] L. G. Apostolova , A. E. Green , S. Babakchanian , et al., “Hippocampal Atrophy and Ventricular Enlargement in Normal Aging, Mild Cognitive Impairment (MCI), and Alzheimer Disease,” Alzheimer Disease and Associated Disorders 26 (2012): 17–27. 22343374 10.1097/WAD.0b013e3182163b62 PMC3286134

[74] E. Cavedo , M. Boccardi , and R. Ganzola , “Local Amygdala Structural Differences With 3T MRI in Patients with Alzheimer Disease,” Neurology 76, no. 8 (2011): 727–733. 21339500 10.1212/WNL.0b013e31820d62d9 PMC3053328

[75] J. P. Li , P. L. Pan , R. Huang , and H. F. Shang , “A Meta‐Analysis of Voxel‐Based Morphometry Studies of White Matter Volume Alterations in Alzheimer's Disease,” Neuroscience and Biobehavioral Reviews 36 (2012): 757–763. 22192882 10.1016/j.neubiorev.2011.12.001

[76] O. Carmichael , C. Schwarz , D. Drucker , et al., “Longitudinal Changes in White Matter Disease and Cognition in the First Year of the Alzheimer Disease Neuroimaging Initiative,” Archives of Neurology 67 (2010): 1370–1378. 21060014 10.1001/archneurol.2010.284 PMC3082636

[77] P. A. Thomann , V. Dos Santos , P. Toro , P. Schönknecht , M. Essig , and J. Schröder , “Reduced Olfactory Bulb and Tract Volume in Early Alzheimer's Disease‐A MRI Study,” Neurobiology of Aging 30 (2009): 838–841. 17875348 10.1016/j.neurobiolaging.2007.08.001

[78] S. L. Risacher , A. J. Saykin , J. D. West , L. Shen , H. A. Firpi , and B. C. Mcdonald , “Baseline MRI Predictors of Conversion From MCI to Probable AD in the ADNI Cohort,” Current Alzheimer Research 6 (2009): 347–361. 19689234 10.2174/156720509788929273 PMC2764863

[79] L. W. De Jong , K. Van Der Hiele , I. M. Veer , et al., “Strongly Reduced Volumes of Putamen and Thalamus in Alzheimer's Disease: An MRI Study,” Brain 131 (2008): 3277–3285. 19022861 10.1093/brain/awn278 PMC2639208

[80] J. L. Whitwell , M. M. Shiung , S. A. Przybelski , et al., “MRI Patterns of Atrophy Associated With Progression to AD in Amnestic Mild Cognitive Impairment,” Neurology 70 (2008): 512–520. 17898323 10.1212/01.wnl.0000280575.77437.a2 PMC2734138

[81] A. Duarte , S. Hayasaka , A. Du , et al., “Volumetric Correlates of Memory and Executive Function in Normal Elderly,” Mild Cognitive Impairment and Alzheimer's Disease 406 (2006): 60–65. 10.1016/j.neulet.2006.07.029 PMC1779764 16904823

[82] C. Pennanen , M. Kivipelto , S. Tuomainen , et al., “Hippocampus and Entorhinal Cortex in Mild Cognitive Impairment and Early AD,” Neurobiology of Aging 25 (2004): 303–310. 15123335 10.1016/S0197-4580(03)00084-8

[83] B. G. Rash , K. N. Ramdas , N. Agafonova , et al., “Allogeneic Mesenchymal Stem Cell Therapy With Laromestrocel in Mild Alzheimer's Disease: A Randomized Controlled Phase 2a Trial,” Nature Medicine 31 (2025): 1257–1266. 10.1038/s41591-025-03559-0 PMC12003194 40065171

[84] S. Abushakra , A. Power , D. Watson et al., “Clinical Efficacy, Safety and Imaging Effects of Oral Valiltramiprosate in APOEε4/ε4 Homozygotes with Early Alzheimer's Disease: Results of the Phase III, Randomized, Double‐Blind, Placebo‐Controlled, 78‐Week APOLLOE4 Trial,” Drugs 85 (2025): 1455–1472. 41015981 10.1007/s40265-025-02250-5 PMC12572085

[85] F. Zhang , Y. Qin , L. Xie , C. Zheng , X. Huang , and M. Zhang , “High‐Frequency Repetitive Transcranial Magnetic Stimulation Combined With Cognitive Training Improves Cognitive Function and Cortical Metabolic Ratios in Alzheimer's Disease,” Journal of Neural Transmission 126 (2019): 1081–1094. 31292734 10.1007/s00702-019-02022-y

[86] J. Penner , R. Rupsingh , M. Smith , J. L. Wells , M. J. Borrie , and R. Bartha , “Increased Glutamate in the Hippocampus After Galantamine Treatment for Alzheimer Disease,” Progress in Neuro‐Psychopharmacology & Biological Psychiatry 34 (2010): 104–110. 19833161 10.1016/j.pnpbp.2009.10.007

[87] P. J. Modrego , M. A. Pina , N. Fayed , and M. Díaz , “Changes in Metabolite Ratios After Treatment With Rivastigmine in Alzheimer's Disease A Nonrandomised Controlled Trial with Magnetic Resonance Spectroscopy,” CNS Drugs 20, no. 10 (2006): 867–877. 16999455 10.2165/00023210-200620100-00006

[88] S. K. Ranga Rama Krishnan , M. H. Cecil Charles , P. Murali Doraiswamy , et al., “Randomized, Placebo‐Controlled Trial of the Effects of Donepezil on Neuronal Markers and Hippocampal Volumes in Alzheimer's Disease,” American Journal of Psychiatry 160, no. 11 (2003): 2003–2011. 14594748 10.1176/appi.ajp.160.11.2003

[89] G. Öz , D. K. Deelchand , J. P. Wijnen , et al., “Advanced Single Voxel 1H Magnetic Resonance Spectroscopy Techniques in Humans: Experts' Consensus Recommendations,” NMR in Biomedicine 34 (2021): e4236. 10.1002/nbm.4236 PMC7347431 31922301

[90] R. S. Jones and A. D. Waldman , “1H‐MRS Evaluation of Metabolism in Alzheimer's Disease and Vascular Dementia,” Neurological Research 26 (2004): 488–495. 15265265 10.1179/016164104225017640

[91] K. Kantarci , C. R. Jack , Y. C. Xu , et al., “Regional Metabolic Patterns in Mild Cognitive Impairment and Alzheimer's Disease A 1 H MRS Study,” Neurology 55 (2000): 210–217. 10908893 10.1212/wnl.55.2.210 PMC2771162

[92] R. Perneczky , Biomarkers for Alzheimer's Disease Drug Development Second Edition, vol. 2785 (Springer US, 2024).

[93] J. Graff‐Radford and K. Kantarci , “Magnetic Resonance Spectroscopy in Alzheimer's Disease,” Neuropsychiatric Disease and Treatment 9 (2013): 687–696. 23696705 10.2147/NDT.S35440 PMC3658533

[94] M. Huang , H. Yu , X. Cai , Y. Zhang , W. Pu , and B. Gao , “A Comparative Study of Posterior Cingulate Metabolism in Patients With Mild Cognitive Impairment due to Parkinson's Disease or Alzheimer's Disease,” Scientific Reports 13 (2023): 13. 37648724 10.1038/s41598-023-41569-5 PMC10469183

[95] R. I. Scahill , J. M. Schott , J. M. Stevens , et al., “Mapping the Evolution of Regional Atrophy in Alzheimer's Disease: Unbiased Analysis of Fluid‐Registered Serial MRI,” PNAS 99 (2002): 4703–4707. 11930016 10.1073/pnas.052587399 PMC123711

[96] K. Kantarci , R. C. Petersen , B. F. Boeve , et al., “1H MR Spectroscopy in Common Dementias,” Neurology 63 (2004): 1393–1398. 15505154 10.1212/01.wnl.0000141849.21256.ac PMC2766798

[97] H. Shinno , T. Inagaki , T. Miyaoka , et al., “A Decrease in N‐Acetylaspartate and an Increase in Myoinositol in the Anterior Cingulate Gyrus Are Associated With Behavioral and Psychological Symptoms in Alzheimer's Disease,” Journal of the Neurological Sciences 260 (2007): 132–138. 17540407 10.1016/j.jns.2007.04.017

[98] F. C. Goldstein , H. Mao , L. Wang , C. Ni , J. J. Lah , and A. I. Levey , “White Matter Integrity and Episodic Memory Performance in Mild Cognitive Impairment: A Diffusion Tensor Imaging Study,” Brain Imaging and Behavior 3 (2009): 132–141. 20596297 10.1007/s11682-008-9055-y PMC2894481

[99] M. A. Ahulló‐Fuster , T. Ortiz , E. Varela‐Donoso , J. Nacher , and M. L. Sánchez‐Sánchez , “The Parietal Lobe in Alzheimer's Disease and Blindness,” Journal of Alzheimer's Disease 89 (2022): 1193–1202. 10.3233/JAD-220498 36093700

[100] I. Apostolova , C. Lange , A. Mäurer , et al., “Hypermetabolism in the Hippocampal Formation of Cognitively Impaired Patients Indicates Detrimental Maladaptation,” Neurobiology of Aging 65 (2018): 41–50. 29407465 10.1016/j.neurobiolaging.2018.01.002

[101] M. De Michele , M. Iacobucci , F. Letteri , et al., “A Transient Magnetic Resonance Spectroscopy Peri‐Ischemic Peak: A Possible Radiological Biomarker of Post‐Stroke Neurogenesis,” Neurological Sciences 44 (2023): 967–978. 36348170 10.1007/s10072-022-06479-w

[102] S. F. Keevil , B. Barbiroli , and J. C. W. Brooks , “Absolute Metabolite Quantification by In Vivo NMR Spectroscopy: II. A Multicentre Trial of Protocols for In Vivo Localised Proton Studies of Human Brain,” Magnetic Resonance Imaging 16, no. 9 (1998): 1093–1106. 9839993 10.1016/s0730-725x(98)00118-0

[103] A. Lin , O. Andronesi , W. Bogner , et al., “Minimum Reporting Standards for in Vivo Magnetic Resonance Spectroscopy (MRSinMRS): Experts' Consensus Recommendations,” NMR in Biomedicine 34 (2021): e4484. 33559967 10.1002/nbm.4484 PMC8647919

[104] C. Dang , Y. Wang , Q. Li , and Y. Lu , “Neuroimaging Modalities in the Detection of Alzheimer's Disease‐Associated Biomarkers,” Review 3 (2023): 1–17. 10.1093/psyrad/kkad009 PMC11003434 38666112

[105] L. Chouliaras and J. T. O'brien , “The Use of Neuroimaging Techniques in the Early and Differential Diagnosis of Dementia,” Expert Review 28 (2003): 4084–4097. 10.1038/s41380-023-02215-8 PMC10827668 37608222

